# MEK5/ERK5 Signaling Suppresses Estrogen Receptor Expression and Promotes Hormone-Independent Tumorigenesis

**DOI:** 10.1371/journal.pone.0069291

**Published:** 2013-08-09

**Authors:** James W. Antoon, Elizabeth C. Martin, Rongye Lai, Virgilo A. Salvo, Yan Tang, Ashley M. Nitzchke, Steven Elliott, Seung Yoon Nam, Wei Xiong, Lyndsay V. Rhodes, Bridgette Collins-Burow, Odile David, Guandi Wang, Bin Shan, Barbara S. Beckman, Kenneth P. Nephew, Matthew E. Burow

**Affiliations:** 1 Department of Pharmacology, Tulane University School of Medicine, New Orleans, Louisiana, United States of America; 2 Department of Medicine, Section of Hematology & Medical Oncology, Tulane University School of Medicine, New Orleans, Louisiana, United States of America; 3 Department of Pathology, Tulane University School of Medicine, New Orleans, Louisiana, United States of America; 4 Department of Pulmonary Diseases, Critical Care, and Environmental Medicine, Tulane University School of Medicine, New Orleans, Louisiana, United States of America; 5 Department of Chemistry, Xavier University, New Orleans, Louisiana, United States of America; 6 Department of Cellular and Integrative Physiology, Indiana University School of Medicine, Bloomington, Indiana, United States of America; Texas A&M University, United States of America

## Abstract

Endocrine resistance and metastatic progression are primary causes of treatment failure in breast cancer. While mitogen activated protein kinases (MAPKs) are known to promote ligand-independent cell growth, the role of the MEK5-ERK5 pathway in the progression of clinical breast carcinoma remains poorly understood. Here, we demonstrated increased ERK5 activation in 30 of 39 (76.9%) clinical tumor samples, as well as across breast cancer cell systems. Overexpression of MEK5 in MCF-7 cells promoted both hormone-dependent and hormone-independent tumorigenesis *in vitro* and *in vivo* and conferred endocrine therapy resistance to previously sensitive breast cancer cells. Expression of MEK5 suppressed estrogen receptor (ER)α, but not ER-β protein levels, and abrogated downstream estrogen response element (ERE) transcriptional activity and ER-mediated gene transcription. Global gene expression changes associated with upregulation of MEK5 included increased activation of ER-α independent growth signaling pathways and promotion of epithelial-to-mesenchymal transition (EMT) markers. Taken together, our findings show that the MEK5-ERK5 pathway mediates progression to an ER(−), mesenchymal and endocrine therapy resistant phenotype. Given the need for new clinical therapeutic targets, our results demonstrate the therapeutic potential of targeting the MEK5-ERK5 pathway in breast cancer.

## Introduction

Despite recent advances in endocrine therapy and the development of new agents, resistance remains a major obstacle in the treatment of breast cancer. The progression of cancer cells to a resistant phenotype is generally characterized by the acquisition of cellular or molecular changes that alter the response to therapeutic agents. Both acquired and *de novo* resistance occurs through enhanced cellular signaling cascades that circumvent estrogen receptor (ER)-dependent proliferation [1]. Resistance is primarily characterized by progression of ER-α(+) cancers to an ER-α(−) phenotype or the acquisition of secondary signaling networks that bypass the requirement for ER-α activity [2]. The loss of ER-α function and expression results in resistance to both primary and secondary endocrine therapeutics, creating a significant deficit of available treatment options. The development of hormone-independence and transition to a mesenchymal phenotype are hallmarks in the progression to endocrine resistance and metastasis [3], [4]. A better understanding of the mechanisms involved in the progression to endocrine resistance is critical for developing new targeted breast cancer therapies.

There is mounting evidence in the literature concerning the role of mitogen activated protein kinases (MAPKs) in cancer development and response to therapeutics. Several reports have demonstrated MAPKs regulate cancer cell survival, anti-apoptotic signaling, angiogenesis, proliferation, and hormone-independence [5], [6], [7], [8], [9], [10], [11]. However, the majority of these studies have focused on the ERK1/2, JNK and p38 families. The MEK5-ERK5 pathway remains the least studied of the MAPK family members. A number of studies have demonstrated overexpression or activation of the MEK5-ERK5 pathway in glioblastoma, leukemia, lymphoma, medulloblastoma, and prostate cancer [12], [13], [14]. While some reports have suggested a role for the MEK5-ERK5 pathway in cancer oncogenesis this pathway's role in breast cancer cells has not been fully explored. MEK5 has been demonstrated to be overexpressed in 50% of breast tumors and a correlation has been found between tumor overexpression of MEK5 and increased activation of STAT3, which is associated with proliferation and metastasis [15], [18]. Additionally, ERK5 has been demonstrated to be overexpressed in 20% of patients and increased expression of ERK5 in breast tumor samples correlated with earlier relapse [16]. These data support recent findings from our laboratory reporting MEK5 overexpression in ER-α (−) breast cancer cells promotes breast cancer therapeutic resistance [17].

Both MEK5 and ERK5 are structurally and functionally distinct from other MAPKs [19], [20], [21]. MEK5 has a novel docking site on the N-terminus containing a different consensus motif than other MEKs [22]. Furthermore, ERK5 contains a larger C-terminus than other MAPKs, which regulates activation, auto-phosphorylation, nuclear transport and subcellular localization of the kinase [23], [24]. It has been speculated that the larger C-terminus of ERK5 may allow for specific targeting by inhibitors without affecting other kinases in the pathway. ERK5 also contains a transcriptional activation domain, suggesting that the enzyme may exert direct kinase activity or induction of gene expression, unlike other ERK kinases [25]. Once in the nucleus, ERK5 can activate several transcription factors including Sap1, c-FOS, c-MYC, and MEF2 [26]. We, along with others, have also demonstrated a role for ERK5 in activation of NF-κB and AP1 mediated gene transcription [27], [28]. However, to date the mechanisms of MEK-ERK5 signaling and its effects on global gene transcription are not wholly understood.

The purpose of this study is to elucidate the role of MEK-ERK5 signaling in the progression of breast cancer. Recent studies have demonstrated a correlation between ER-α expression, ERK1/2 signaling, and hormone independence [8], [9], [10], [11]. Yet, the role of MEK5 signaling in the regulation of ER-α expression and progression to hormone independence is unclear. Defining the mechanisms of MEK5-ERK5 signaling in the regulation of ER-α expression and EMT will significantly impact our understanding of tumor progression and clinical drug resistance. A better understanding of MEK5-ERK5 and subsequent downstream signaling may lead to new therapeutic targets in the treatment of endocrine resistant breast cancer.

## Materials and Methods

### Ethics Statement

All procedures involving animals were conducted in compliance with State and Federal laws, standards of the U.S. Department of Health and Human Services, and guidelines established by the Tulane University Animal Care and Use Committee. The facilities and laboratory animal program of Tulane University are accredited by the Association for the Assessment and Accreditation of Laboratory Animal Care. The Tulane institutional review board approved the use of animals and human tissues in this study. The Tulane institutional review board approved the use of human tissue samples and written informed consent for the original human work that produced the tissue samples.

### Patient Samples Staining and Analysis

Breast slides (T-BO-1) were obtained from the tissue array research program (TARP) (National Cancer Institute and National Human Genome Research Institute). Thirty-nine infiltrating breast carcinomas were represented in the array. Immuno-staining for phospho-ERK5 was performed in house and the results were examined using an immuno-histochemical (IHC) histologic score (H-score) incorporating intensity and distribution of staining. The H-score is described by: HS 1/4 (p*i)/100, where p denotes the percentage of stained cells and i denotes the intensity of the staining [29]. The H-score scale was 0–3. Staining scale: 0, none; 1, weak; 2, moderate; and 3, strong. Scoring was performed blinded by trained pathologists [30]. A tumor was scored as 0 if there were no appreciable staining in tumor cells compared with stromal elements, as 1 if there were barely detectable staining in cytoplasm and/or nucleus compared with stromal elements, as 2 if there were readily appreciable brown staining distinctly marking tumor cell cytoplasm and/or nucleus, and as 3 if there was dark brown staining in tumor cells completely obscuring cytoplasm and/or nucleus.

### Immunohistochemistry of Xenograft Tissue

Immuno-staining was performed as previously described [31], [32]. Briefly, slides from tumors were deparaffinized and rehydrated. For antigen retrieval sections were heated for 25 minutes at 95°C in the presence of Rodent decloaker. Samples were then allowed to cool for 20 minutes at room temperature. Slides were then blocked in rodent block for 30 minutes and then with primary PgR and ER-α antibodies for one hour. Mouse-on-mouse HRP-polymer secondary antibody was added to the sections for 15 minutes at room temperature. Subsequently, slides were incubated in DAB for one minute and then counterstained for 30 seconds. A Leica DM IRB Inverted Research microscope and SPOT RT color camera were used to view slides; original magnification at 400×. For staining quantification numbers of positively stained cells were represented as percentage of total number of cells per field of view.

### Cells and Reagents

MCF-7N cell variant (subclone of MCF-7 human breast adenocarcinoma line from American Type Culture Collection (ATCC)) was generously provided by Louise Nutter (University of Minnesota, Minneapolis, MN) in 1996 [33]. The MCF-7-MEK5 cells (MCF-7 cells stably overexpressing MEK5) were generated as previously described [34]. The breast cancer cell lines MDA-MB-231, MDA-MB-361 (ER-α (−)), ZR75,T47D, andSKBR3 were acquired from ATCC in 2004. Liquid nitrogen stocks were made upon receipt and maintained until the start of each study. Estrogen response element–luciferase and/or qPCR for ER and progesterone receptor (PgR) were used to confirm cell lines sustained estrogen responsiveness. Morphology and doubling times were also recorded regularly to ensure maintenance of phenotype. Cells were used for no more than 6 months after being thawed. Cells were cultured as previously described [35]. ICI 182,780 was purchased from Tocris Bioscience (Ellisville, MO). Dimethylsulfoxide (DMSO) and 17β-estradiol (E2, estrogen) were purchased from Fisher Scientific (Waltham, MA). 4-Hydroxytamoxifen (tamoxifen, OHT) was purchased from Sigma-Aldrich (St. Louis, MO). Dosing for these reagents was E2 (1 nM), tamoxifen (100 nM) and ICI 182, 780 (100 nM) unless otherwise indicated.

### Western Blot Analysis

Western blot analyses were conducted as published [35]. Cells were maintained in 10% FBS DMEM for 24 hours prior to harvesting for protein extraction. Membranes were probed with primary antibodies according to manufacturer's protocol. Antibodies: ER-β, APγ, and β-actin were purchased from Cell Signaling (Danvers, MA) (dilution 1∶1000) and total ER-α, ERK5, and GAPDH were purchased from Santa Cruz Biotechnology (Dallas, Texas) (dilution 1∶250). IR-tagged secondary antibodies were purchased from LiCor Biosciences (Lincoln, Nebraska). Blots were analyzed by the Odyssey Infrared Imaging System (LiCor Biosciences). Experiments were conducted in triplicate with representative blots shown.

### Animal Studies

Xenograft tumor studies were conducted as previously described [32], [35]. Immune-compromised female ovariectomized mice (29–32 days old) were obtained from Charles River Laboratories (Wilmington, MA). The animals were allowed a period of adaptation in a sterile and pathogen-free environment with food and water *ad libitum*. When stated, placebo or E2 pellets (0.72 mg, 60-day release; Innovative Research of America, Sarasota, FL) were implanted subcutaneously in the lateral area of the neck in the middle point between the ear and shoulder using a precision trochar (10 gauge). MCF-7-vector, MCF-7-MEK5, MCF-7-MEK5-(empty shRNA), MCF-7-MEK5-(ERK5 shRNA) cells were harvested and viable cells mixed with Matrigel Reduced Factors (BD Biosciences, San Jose, CA). Injections (5×10^6^cells/injection) were made bilaterally into the mammary fat pad. All the procedures in animals were carried out under anesthesia using a mix of isoflurane and oxygen delivered by mask. Tumor size was measured every 2 days using a digital caliper. The volume of the tumor was calculated using the following formula: 4/3πLM^2^, where L is the larger radius and M is the smaller radius. At necropsy animals were euthanized by cervical dislocation after CO_2_ exposure. Tumors were removed and either frozen in liquid nitrogen or fixed in 10% formalin for further analysis.

### Microarray Data Analysis and Validation

MCF-7-vector and MCF-7-MEK5 cell lines were grown in DMEM supplemented with 10% fetal bovine serum (FBS) (10% DMEM) for 24 hours prior to extraction for microarray analysis. Microarray analysis was performed according to previously published protocols [36]. The hybridized Human Genome U133A 2.0 Array was scanned and analyzed using the Affymetrix Microarray Analysis Suite version 5.0. The average density of hybridization signals from four independent samples was used for data analysis and genes with signal density <300 pixels were omitted from the data analysis. P-values were calculated with two-sided t-tests with unequal variance assumptions, and a p-value of <0.001 was considered to be significant. The fold-change was described as a positive value when the expression level was increased and a negative value when the expression level was reduced. False discovery rate (FDR) was set at 0.1 in the data analysis. To confirm the gene expression data from microarray analysis, quantitative PCR was used to examine the mRNA levels of a subset of genes. The quantitative PCR results showed a high degree of correlation to the microarray data.

### RNA Isolation and Quantitative Real-Time PCR

RNA Isolation and qRT-PCR was performed as previously described [36]. Briefly, RNA was isolated from cultured cells using RNeasy kit as per manufacture's protocol (Qiagen, Germantown, MD) and evaluated spectrophotometrically by absorbance (260, 280 nm). 1 µg total RNA was reverse-transcribed (iScript kit; BioRad, Hercules, CA) as previously published [36]. Primer sequences are available in Supplementary Materials and Methods. Data analyses compare relative target expression to β-actin control and relative gene expression was analyzed using the 2^ΔΔCt^ method (24). Treatments and time points are specified in the figure legends. Experiments were conducted in triplicate.

### Estrogen Response Element (ERE)-Luciferase Assay

As previously described [32], the cells were seeded in 24-well plates at a density of 50,000 cells per well in 5% charcoal/dextran treated FBS DMEM and allowed to attach overnight. After 18 h, cells were transfected with 300 ng pGL2- ERE2X-TK-luciferase plasmid, using 6 µl Effectene (Qiagen) per microgram of DNA. After 5 hours, cells were treated with vehicle or E2 and incubated at 37°C. After 18 hours, the medium was removed, and 100 µl lysis buffer was added per well and then incubated for 15 min at room temperature. Luciferase activity for the cell extracts was determined using luciferase substrate (Promega Corp., Madison, WI) in an Autoluminat Plus luminometer (Berthhold Technologies, Bad Wildbad, Germany).

### Cell Viability Assay

Viability assays were performed as previously described [32]. Briefly, cells were plated at a density of 7,500 cells per well in a 96-well plate in phenol-free DMEM supplemented with 5% charcoal/dextran treated FBS (5% CS-DMEM) and allowed to attach overnight. Cells were then treated with ICI 182,780 or tamoxifen for 24 hours. After treatment, 20 µl 3-(4,5-dimethylthiazol-2-yl)-2,5-diphenyltetrazolium bromide (MTT, 5 mg/ml) reagent was incubated in each well for 4 hours. Cells were lysed with 20% sodium dodecyl sulfate (SDS) in 50% dimethylformamide. The pH and absorbance values were read on an EL×808 Microtek plate reader (Bio-Tek Instruments, Winooski, VT) at 550 nm, with a reference wave- length of 630 nm.

### Clonogenic Survival Assay

Colony assays were performed as described in previously published methods [37]. Cells were serum starved for 24 hours prior to being plated in 6-well plates at a density of 1,000 cells per well in 5% CS-DMEM. 24 hours later cells were treated with indicated concentrations of vehicle, ICI 182,780, or tamoxifen and then monitored for colony growth. Ten days later the cells were fixed with 3% glutaraldehyde for 15 minutes. Following fixation, the plates were washed and stained with a 0.4% solution of crystal violet in 20% methanol for 30 minutes, washed with PBS, and dried. Colonies of ≥50 cells were counted as positive. Results were normalized to DMSO (vehicle) treated cells.

### RNA Interference

shRNA transfections were performed using Fugene6 as previously described [38]. ERK5-specific shRNA (SureSilencing shRNA) and control shRNA vector plasmids have been previously described [34]. MCF-7-MEK5 cells were grown in a 100 mm dish. Following transfection cells were treated with 300 ng/ml puromycin. Cells were grown in 10% FBS DMEM and treated with 300 ng/ul puromycin every two days for 2 weeks. Colonies were pooled and verification of ERK5 suppression was confirmed using RT-PCR and western blot. Stable pools were maintained in 10% FBS DMEM as described above.

### Reverse transcriptase PCR

RT-PCR was performed as previously described [36]. RNA was isolated from cultured cells using RNeasy (Qiagen) and evaluated spectrophotometrically by absorbance (260, 280 nm). Two micrograms of cDNA was transcribed with SuperScript III (Invitrogen, Grand Island, NY) and mRNA was amplified. Primers were used at 20 nmol/L final concentration. Primer sequences are available upon request.

### Transient Transfection of AP2γ

MCF-7-MEK5 cells were plated at 1×10^6^ cells per dish and allowed to adhere over night in 10% FBS DMEM. 5 ug of pCDNA-vector or AP2γ (Origene, Rockville, MD) were transiently transfected into each cell line using Lipofectamine (Invitrogen). Cells were then harvested for qPCR for validation of AP2γ and ER-α levels.

### Statistical Analysis

Studies involving more than 2 groups were analyzed by 1-way ANOVA with Tukey's post-test; all others were subjected to unpaired Student's t-test (GraphPad Prism V.4) as previously described [36], [39], [40]. For pathway analysis, data processing and statistics were carried out as we have described [41]. Using Bioconductor, present (P), absent (A) or marginal (M) calls were determined using an MAS5 algorithm. Fraction presence, defined as the average present/absent (P/A) detection call (scores were given as P = 1, M = 0.5 and A = 0) for the groups, was calculated for each microarray probe, and probes with at least one group having a fraction presence of 0.5 were selected. Welch's t-test was performed for each probe using their log-transformed signals, with p-values less than 0.01 considered significant. To further support the statistical significance of probes having p<0.01, the FDR was also calculated with probe significance defined as an FDR of less than 5%. A moderately stringent fold-change cutoff of ≥2.0 (or ≤−2.0 for down-regulation), which allows for an acceptable balance between false discovery and false-negative rates was applied (in addition to the p-value cutoffs of p<0.001) to determine genes with significant expression alterations.

## Results

### ERK5 Activation in Clinical Breast Carcinoma and Breast Cancer Cell Lines

**Figure 1:**
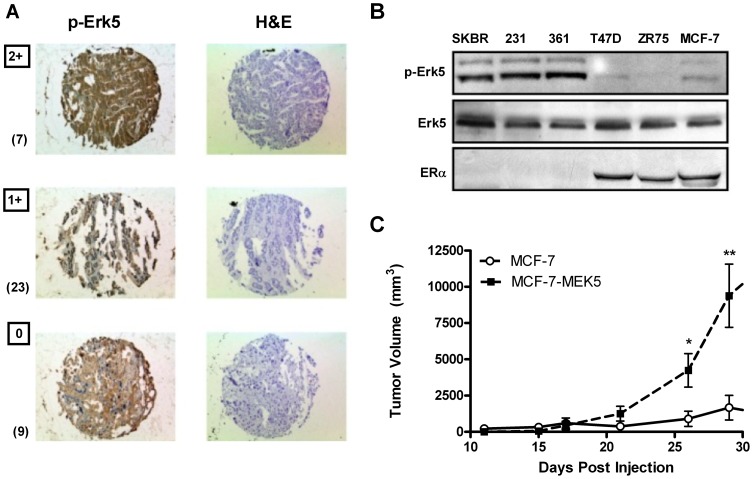
Phosphorylated ERK5 in human breast carcinoma. (A) Thirty nine human breast carcinoma tissue samples were stained with anti-p-ERK5 (Thr218/Tyr220) and H&E, results demonstrate representative samples with histological score (H-score) 0–2. (B) Western Blot for protein expression levels of phospho- and total ERK5 and ER-α across breast cancer cell lines. (C) MCF-7 cells expressing either MEK5 (MCF7-MEK5) or empty vector were injected into the mammary fat pad of Nu/Nu mice in the presence of E2 pellets (0.76 mg, 60 day release) (n = 10/group). Points represent mean tumor volume ± SEM; * significantly different from vector p<0.05; ** significantly different from vector p<0.01.

While total MEK5 and ERK5 protein expressions in patients has been reported at 50% and 20% respectively, the prevalence of phospho-ERK5 in clinical breast cancer has not yet been investigated by immuno-histochemistry. Therefore, we first determined the relevance of ERK5 activation in clinical breast tumors. To obtain clinical correlation, tissue arrays of 39 unidentified patient samples were analyzed for phospho-state specific antibodies to the Thr218/Tyr220 activation site of ERK5. The array slides were then scored by a pathologist by assessing the intensity of tumor cell staining relative to stromal elements in the same spot. Results demonstrate expression of phospho-ERK5 in 30 of 39 (76.9%) of infiltrating breast cancer biopsy samples (Fig. 1A). Similar ERK5 activation profiles were also found in invasive ovarian cancer tissue arrays using the same scoring system (Fig. S1). These results suggest that ERK5 pathway is a clinically relevant cancer signaling pathway.

We next determined if well established breast cancer cell lines also exhibited activation of ERK5. Phosphorylated ERK5 levels at the Thr218/Tyr220 activation site were examined by Western blot analysis in human breast cancer cell lines with varying degrees of ER-α expression. The MCF-7 cells, T47D and ZR-75 cells are models of ER-α (+) breast carcinoma whereas SKBR3 and MDA-MB-231 cells are well-established ER-α (−) breast cancer models. Variants of MDA-MB-361 exist, and here we use MDA-MB-361 cells that are ER-α (−) [42]. Analysis revealed increased phospho-ERK5 protein levels in the ER-α (−) SKBR3, MDA-MB-231 and MDA-MB-361 cell lines (Fig. 1B). These results were in stark contrast to the ER-α (+) cell lines MCF-7, T47D and ZR-75, which displayed markedly low phopho-ERK5 levels, demonstrating an inverse correlation between phospho-ERK5 protein expression and ER-α levels.

Our laboratory recently generated a stable constitutively active MEK5 overexpressing cell line (MCF-7-MEK5) from parental ER-α (+) MCF-7 cells [34]. We utilized this cell model to examine the effect of MEK5 signaling on breast cancer tumor growth. Using a NOD/SCID murine model of *in vivo* tumorigenesis, tumor formation of MCF-7-MEK5 overexpressed xenografts was compared to MCF-7-vector cells in the presence of estrogen. Results demonstrate earlier tumor initiation in the MEK5 expressing cells (p<0.05) and greater tumor growth (p<0.05) compared to MCF-7-vector tumors (Fig. 1C).

### Global Gene Expression Profiles Associated with MEK5 Overexpression

**Figure 2:**
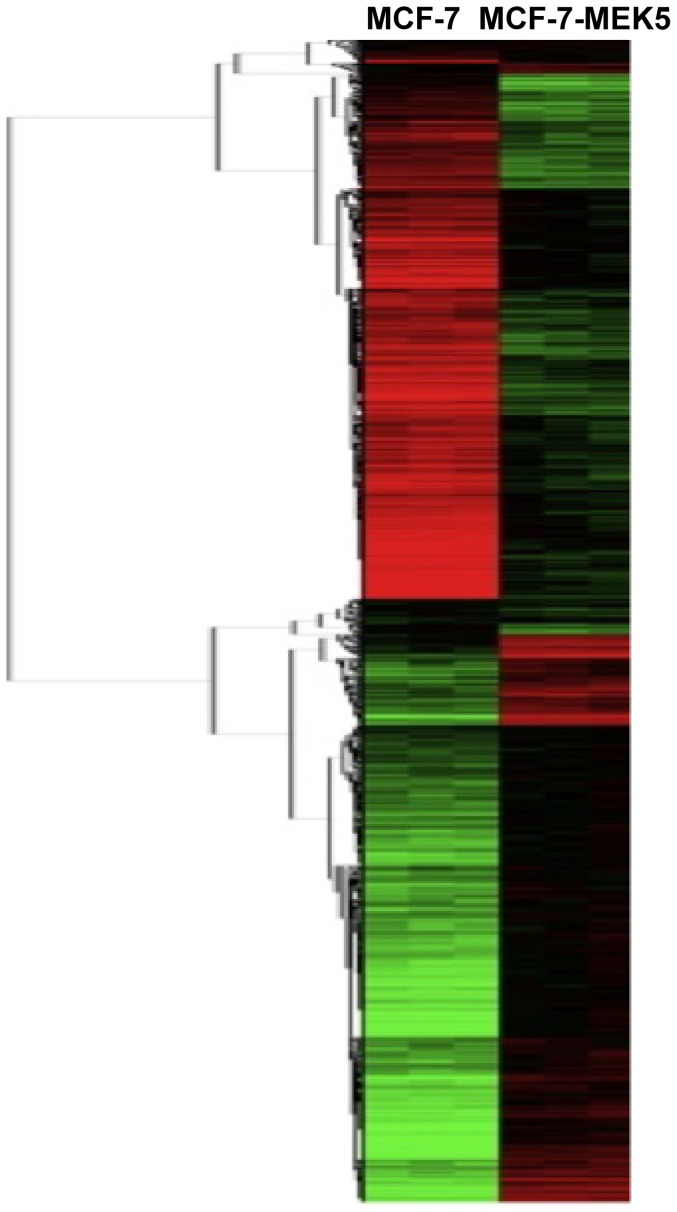
Clustering analysis of mRNA expression profiles of MCF- 7 and MCF-7-MEK5 cells. Microarray results demonstrating MCF-7 (left column) and MCF-7-MEK5 (right column) cell lines have distinctive gene expression patterns, with samples of same cell lines clustered together. Trees on the left are gene clusters. Data represented as mean ± S.E.M. of 3 independent experiments.

We next sought to investigate the mechanism of the observed MEK5 mediated tumorigenesis. Global gene expression profiling was performed on MCF-7-MEK5 cells and compared to MCF-7-vector cells (Fig. 2). The above analysis identified 3404 significantly altered genes: 1883 up-regulated and 1521 down-regulated transcripts. Although the altered genetic profile in MEK5 overexpression was diverse, it could be organized into functional signaling categories using the Kyoto Encyclopedia of Genes and Genomes database and Gene Ontology algorithms. Analysis revealed several pathways significantly altered in the MCF-7-MEK5 overexpressing cells. Interestingly, some of these pathways are known to regulate cancer signaling in other tumor types, including leukemia, lymphoma, melanoma, and prostate (Table 1). These results suggest that the MEK5 pathway is a relevant signaling pathway in various cancers in addition to breast.

**Table 1:** Cancer Signaling Pathways Associated with MEK5.

Pathway Name	Impact Factor	Number of Pathway Genes	Genes Altered	Percent Pathway Genes Altered	p-value
Leukocyte Transendothelial Migration	287.70	119	24	20.17	2.52E-02
Cell Adhesion Molecules	185.12	134	32	23.88	6.96E-04
Adherens Junction	25.78	78	23	29.49	1.56E-04
Axon Guidance	23.26	129	43	33.33	5.25E-09
Pathways in Cancer	19.12	330	81	24.55	2.94E-08
Focal Adhesion	14.29	203	51	25.12	4.96E-06
MAPK Signaling	13.91	272	63	23.16	7.53E-06
ECM-Receptor Interaction	12.58	84	26	30.95	2.37E-05
Tight Junction	12.56	135	35	25.93	7.19E-05
Regulation of Actin Cytoskeleton	12.33	217	52	23.96	1.69E-05
Small Cell Lung Cancer	12.24	86	26	30.23	3.74E-05
ErbB Signaling	10.54	87	19	21.84	2.02E-02
Melanoma	10.39	71	19	26.76	2.03E-03
p53 Signaling	10.16	69	21	30.44	1.82E-04
Pancreatic Cancer	9.66	72	20	27.78	9.50E-04
Non-Small Cell Lung Cancer	8.89	54	16	29.63	1.42E-03
Glioma	8.83	65	18	27.69	1.74E-03
Apoptosis	8.79	89	23	25.84	1.22E-03
Phosphatidylinositol Signaling	7.98	76	20	26.32	1.95E-03
Colorectal Cancer	7.83	84	20	23.81	6.71E-03
Fc epsilon RI Pathway	7.57	78	20	25.64	2.73E-03
Gap Junctions	7.56	96	21	21.88	1.49E-02
Prostate Cancer	7.52	90	21	23.33	7.10E-03
Chronic Myeloid Leukemia	7.12	75	18	24.00	9.00E-03
Bladder Cancer	6.96	42	11	26.19	1.97E-02
Acute Myeloid Leukemia	6.90	59	14	23.73	2.18E-02
Circadian Rhythm	6.76	13	5	38.46	2.18E-02
B cell Receptor Signaling	5.98	65	14	21.54	4.67E-02
VEGF Signaling	5.77	74	16	21.62	3.40E-02
Adipocytokine Signaling	5.63	67	15	22.39	2.96E-02
PPAR Signaling	5.42	70	17	24.29	9.70E-03
Cell Cycle	5.39	118	25	21.19	1.40E-02
ABC Transporters	5.12	44	11	25.00	2.75E-02
Hedgehog Signaling	4.22	57	13	22.81	3.57E-02

The microarray findings were then analyzed to determine the effect of MEK5 activity on breast cancer signaling pathways (Table 2, Table S1). Analysis revealed that MEK5 expression was associated with alterations in signaling pathways involved in oncogenesis and survival, including p53, cell cycle, apoptosis, and gene transcription signaling (Table S1). The changes in gene transcription included those genes known to be mediated by MEK5, including the CREB, MEF, and the AP1 family of transcription factors. Further analysis revealed altered genomic transcription of ER-α independent growth signaling pathways. We found significant changes in gene transcription in the ER, EMT, PI3K/AKT, MAPK, TGF-β and FGF-FGFR signaling pathways (Table 2, Table S1). Alterations in these pathways were consistent with increased tumor growth in MEK5 cells compared to vector.

**Table 2:** Pathway Analysis of MEK5 Mediated Gene Expression Changes.

Gene Symbol	Genbank	Basal Expression (vs MCF-7)	Description
*Cell Adhesion Molecules*			
CDH3	NM_001793.4	−6.16	cadherin 3, type 1, P-cadherin
CLDN3	NM_001306.3	−6.27	claudin 3
CLDN4	NM_001305.3	−9.78	claudin 4
CLDN7	NM_001185022.1	−21.86	claudin 7
CNTN1	NM_001256063.1	25.80	contactin 1
HLA-DPA1	NM_001242525.1	20.11	major histocompatibility complex, class II, DP alpha 1
ITGA4	NM_000885.4	22.64	integrin, alpha 4
ITGA8	NM_003638.1	15.13	ITGA8 integrin, alpha 8
JAM3	NM_001205329.1	15.21	JAM3 junctional adhesion molecule 3
NCAM1	NM_000615.6	10.45	neural cell adhesion molecule 1
NCAM2	NM_004540.3	−5.79	neural cell adhesion molecule 2
NLGN1	NM_014932.2	15.25	neuroligin 1
PTPRM	NM_001105244.1	9.04	protein tyrosine phosphatase, receptor type, M
PVRL3	NM_001243286.1	18.30	poliovirus receptor-related protein 3
SDC4	NM_002999.3	−4.72	syndecan 4
VCAN	NM_001126336.2	54.86	ersican
*Cell Cycle*			
CCND1	NM_053056.2	−7.60	cyclin D1
CDC7	NM_001134419.1	3.30	cell division cycle 7 homolog
SFN	NM_006142.3	−10.89	Stratifin
SKP2	NM_001243120.1	5.16	S-phase kinase-associated protein 2 (p45)
TGFB2	NM_001135599.2	−4.55	transforming growth factor, beta 2
*Epithelial-to-Mesenchymal Transition*			
CDH1	NM_004360.3	−46.38	cadherin 1, type 1, E-cadherin (epithelial)
CDH2	NM_001792.3	48.25	cadherin 2, type 1, N-cadherin (neuronal)
LEF-1	NM_001130713.2	8.68	lymphoid enhancer-binding factor 1
SDC1	NM_001006946.1	−3.60	syndecan 1
SNAI2	NM_003068.4	11.10	snail homolog 2
TWIST1	NM_000474.3	2.77	twist homolog 1
VIM	NM_003380.3	33.99	vimentin
ZEB1	NM_001128128.2	12.29	zinc finger E-box binding homeobox 1
ZEB2	NM_001171653.1	8.74	zinc finger E-box binding homeobox 2
*ErbB Signlaing*			
AREG	NM_001657.2	−134.28	amphiregulin
BTC	NM_001729.2	−3.65	betacellulin
CAMK2D	NM_001221.3	3.47	calcium/calmodulin-dependent protein kinase II delta
ERBB3	NM_001005915.1	−5.17	v-erb-b2 erythroblastic leukemia viral oncogene homolog 3
ERBB4	NM_001042599.1	3.04	ERBB4 v-erb-a erythroblastic leukemia viral oncogene homolog 4
PAK1	NM_001128620.1	5.16	p21 protein (Cdc42/Rac)-activated kinase 1
RPS6KB1	NM_003161.2	−3.75	ribosomal protein S6 kinase
SHC4	NM_203349.3	−3.80	SHC (Src homology 2 domain containing) family, member 4
TGFA	NM_001099691.2	−4.91	transforming growth factor, alpha
*Estrogen Receptor*			
TFAP2C	NM_003222.3	−13.56	transcription factor AP-2 gamma
BCAS1	NM_003657.2	−12.13	breast carcinoma amplified sequence 1
CXCL12	NM_000609.5	−5.37	chemokine (C-X-C motif) ligand 12
ESR1	NM_000125.3	−31.25	estrogen receptor isoform 1
GREB1	NM_014668.3	−24.88	growth regulation by estrogen in breast cancer 1
NCOA3	NM_001174087.1	−10.07	nuclear receptor coactivator 3
PGR	NM_000926.4	−11.56	progesterone receptor
PRLR	NM_000949.5	−32.25	prolactin receptor
TFF1	NM_003225.2	−192.11	trefoil factor 1
*Mitogen Activated Protein Kinase Signaling*			
AKT3	NM_001206729.1	86.28	v-akt murine thymoma viral oncogene homolog 3
CACNA2D1	NM_000722.2	28.01	calcium channel, voltage-dependent, alpha 2/delta subunit 1
CACNA2D3	NM_018398.2	9.17	calcium channel, voltage-dependent, alpha 2/delta subunit 3
CACNB2	NM_000724.3	4.23	calcium channel, voltage-dependent, beta 2 subunit
FGF9	NM_002010.2	7.09	fibroblast growth factor 9 (glia-activating factor)
MAP2K6	NM_002758.3	4.73	mitogen-activated protein kinase kinase 6
PLA2G4A	NM_024420.2	14.51	phospholipase A2, group IVA (cytosolic, calcium-dependent)
RASGRF2	NM_006909.2	7.97	Ras protein-specific guanine nucleotide-releasing factor 2
DUSP4	NM_001394.6	−5.56	dual specificity phosphatase 4
HSPB1	NM_001540.3	−6.39	heat shock 27 kDa protein 1
CACNA1D	NM_000720.2	−5.87	CACNA1D calcium channel, voltage-dependent, L type, alpha 1D subunit
CACNG4	NM_014405.3	−8.68	calcium channel, voltage-dependent, gamma subunit 4
*p53 Signaling*			
CCNG1	NM_004060.3	3.08	cyclin G1
CDK6	NM_001145306.1	6.74	cyclin-dependent kinase 6
CDKN2A	NM_000077.4	10.62	cyclin-dependent kinase inhibitor 2A
PMAIP1	NM_021127.2	6.66	phorbol-12-myristate-13-acetate-induced protein 1
PPM1D	NM_003620.3	−3.20	protein phosphatase, Mg2+/Mn2+ dependent, 1D [
SESN1	NM_001199933.1	4.08	sestrin 1
SESN3	NM_144665.2	7.29	sestrin 3
THBS1	NM_003246.2	−45.05	thrombospondin 1
*Gene Transcription*			
TFAP2C	NM_003222.3	−13.5	transcription factor AP-2 gamma
FOS	NM_005252.3	−12.63	FBJ murine osteosarcoma viral oncogene homolog
FOSB	NM_001114171.1	−1.71	FBJ murine osteosarcoma viral oncogene homolog B
TFAP2A	NM_001032280.2	1.87	transcription factor AP-2 alpha
FOSL2	NM_005253.3	2.66	FOS-like antigen 2
JUN	NM_002228.3	2.66	jun proto-oncogene
JUNB	NM_002229.2	8.84	jun B proto-oncogene
MEF2C	NM_001131005.2	21.70	myocyte enhancer factor 2C

### MEK5 Expression Is Associated With Epithelial-Mesenchymal Transition (EMT) Markers

As the analysis above strongly indicated an increase in EMT-associated genes in MEK5 cells, we further examined the microarray data for differences in the expression levels of 168 genes known to promote EMT in breast cancer. The results were similar to the clustering result using the whole mRNA profiles (Fig. S2). The EMT gene expression profile was markedly altered in MCF-7-MEK5 compared to MCF-7-vector cells (Table S2). The EMT gene expression changes in the MEK5 cells are more consistent with a mesenchymal rather than epithelial phenotype. This included loss of E-cadherin and an up-regulation of vimentin, N-cadherin and FGF9 in the MEK5 cells. Consistent with our previously published findings, we observed an up-regulation of ZEB1, ZEB2 and SNAI2 in the MEK5 cells [34]. Other known EMT regulating factors that were increased included Goosecoid, LEF-1 and MMP2. There were no differences in FOXC2 or SNAI1 between the MCF-7-vector and MCF-7-MEK5 cells, but differential expression of Twist was observed. Interestingly, there were also significant changes in the TNF pathway (Table S3). These results are also consistent with our previous findings of MEK5 induced progression to a mesenchymal phenotype [34].

### MEK5 Expression Alters Estrogen Receptor Signaling and Promotes Endocrine Therapy Resistance

EMT has been associated with the loss of ER-α expression [43], [44]. Given the enhanced EMT-associated changes found in our array data and our findings of an inverse correlation between ERK5 activation and ER-α expression (Fig. 1B), we further investigated the ER-α signaling changes associated with MEK5 overexpression. To investigate ER-α genomic activity, clustering analysis was performed on 89 known ER-α mediated genes from our microarray data. Results of this analysis were similar to clustering using the whole mRNA profiles (Fig. S3). MCF-7-MEK5 expressing cells displayed a marked decrease in ER-α gene expression, as well as downstream ER-α regulated gene expression, compared to MCF-7-vector cells (Table S4). This was accompanied by loss of the critical ER-α cofactors NCOA3 and GATA3 and suppression of classic estrogen responsive genes, including PgRSDF-1/CXCL12, GREB1, and prolactin receptor (PRLR) (Table 2 and Table S4).

**Figure 3:**
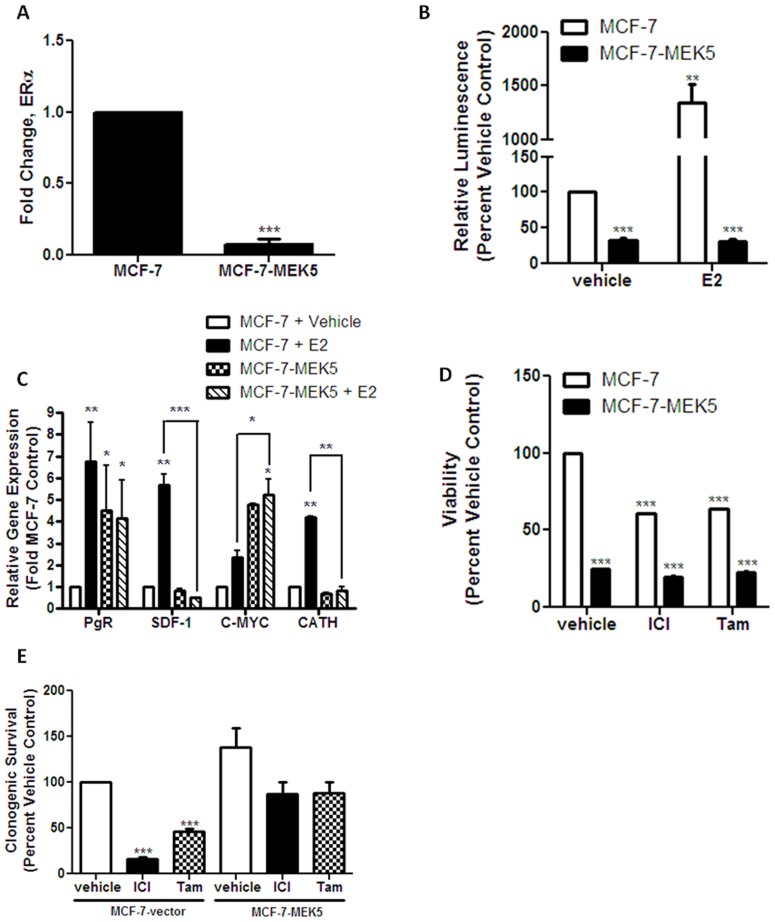
MEK5 expression decreases ER signaling and confers endocrine resistance. (A) MCF-7-vector and MCF-7-MEK5 cells were harvested for total RNA isolation and expression of ER-α mRNA determined using qRT-PCR. Data is represented as mean fold stimulated gene expression normalized to β-actin and vector cells designated as 1. Error bars represent SEM, n = 3. (B) ERE luciferase for MCF-7-vector and –MEK5 cells transiently transfected with pGL2-ERE2X-TK-luciferase plasmid and treated with E2 or vehicle for. Results represent normalized luminescence. Normalization was to vector treated with vehicle and designated as 100 (C) qRT-PCR for MCF-7-vector and –Mek5 cells was performed for E2 responsive genes PgR, SDF-1, c-MYC and Cathepsin-D following E2 treatment. Data is represented as mean fold stimulated gene expression normalized to vehicle treated MCF-7-vcetor cells designnated as 1. Error bars represent SEM, n = 3 independent experiments. (D) MTT ana;ysis of MCF-7-vector and –Mek5 cells following treatment with indicated concentrations of vehicle, ICI or Tam for 24 hours. Data are presented as mean percentage of vehicle treated samples with vehicle normalized to 100. Error bars represent S.E.M., n = 4 independent experiment. (E) Colony assay for MCF-7-vector and –Mek5 cells following treatment with DMSO (vehicle), ICI or Tam. Cells were allowed to grow for 10 days. Colonies of >50 were counted as positive. Results were normalized to percent clonogenic survival of vehicle control cells. Data represented as mean ± S.E.M., n = 4 independent experiments. *, P<0.05; **, P<0.01; ***, P<0.001.

The above findings of decreased ER-α expression were confirmed using qRT-PCR analysis of select genes in the ER signaling pathway. We found significantly decreased ER-α mRNA expression in MEK5 cells compared to vector cells (Fig. 3A). Down-regulation of ER-α in these cells was confirmed using analysis of estrogen response element (ERE) luciferase. MCF-7-MEK5 cells exhibited a diminished estrogen induced ERE transcriptional activity compared to vector cells both at the basal level and following stimulation with E2 (Fig. 3B). To further validate these findings, we determined whether the differential ER-α signaling in MEK5 cells translated to changes in estrogen-induced gene expression. Consistent with the loss of ER-α expression, the MCF-7-MEK5 cells displayed a loss of estrogen-stimulated mRNA expression of PgR, SDF-1, c-Myc, and Cathepsin-D compared to MCF-7-vector cells (Fig. 3C).

The loss of ER-α expression in MCF-7-MEK5 expressing cells parallels observations in clinical breast carcinoma progression to therapeutic resistance [2]. Given the above inverse correlation of phopho-ERK5 activation and decreased ER-α expression in breast cancer cell lines (Fig. 1B) we set out to determine if MEK5-ERK5 signaling could play a role in the progression to endocrine therapy resistance. MCF-7-MEK5 and MCF-7-vector cells were treated with the clinical endocrine therapeutics fulvestrant (ICI 182,780) and tamoxifen and analyzed for cell viability. As seen in Fig. 3D, MTT analysis demonstrates MEK5 overexpression led to an endocrine therapy resistant phenotype, with MCF-7-MEK5 cells exhibiting increased resistance to both ICI 182,780 and tamoxifen compared to MCF-7-vector. MEK5-induced endocrine resistance was confirmed using long-term colony formation assays. Expression of MEK5 increased clonogenic survival following treatment with endocrine therapy (Fig. 3E). Taken together, these results confirmed our microarray findings that MEK5 expression was associated with loss of ER-α activity and suggest progression towards endocrine resistance.

### MEK5-ERK5 Signaling Promotes Progression to an ER-Negative and Hormone Independent Phenotype *In Vivo*

**Figure 4:**
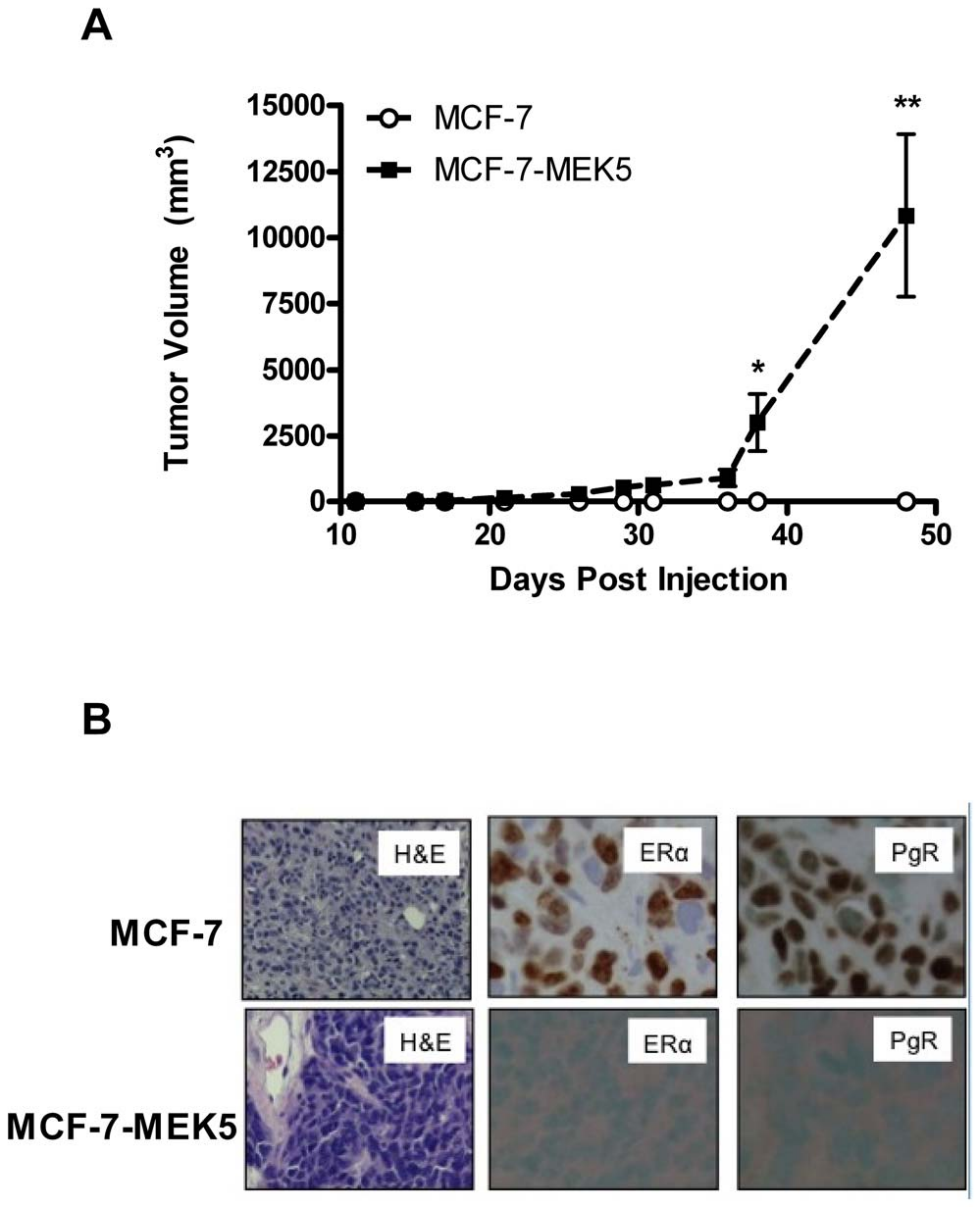
MEK5 expression enhances hormone-independent tumorigenesis. (A) MEK5 (MCF7-MEK5) or vector cells were injected into the mammary fat pad of Nu/Nu mice. Tumor growth was monitored biweekly after palpable tumor formation (n = 10/group). Points represent mean TV ± SEM; *, P<0.05; **, P<0.01. (B) Endpoint tumors from xenograft model of MCF-7-vector and MCF-7-MEK5 treated with E2 were harvested and processed for H&E staining or immunohistochemistry for ER-α or PgR levels.

The above findings of decreased ER-α signaling (Fig. 3) and increased ER-α independent growth signaling (Table S2) were indicative of a hormone independent phenotype in the MCF-7-MEK5 cell line. Therefore, we next sought to confirm this finding of MCF-7-MEK5 hormone-independence in xenograft animal models. MCF-7-vector and MCF-7-MEK5 cells were implanted into the mammary fat pat of Nu/Nu mice in the absence of estrogen and monitored for tumor formation. MEK5 overexpressing cells were capable of tumor formation while MCF-7-vector cells were unable to form tumors without estrogen as far out as 50 days (Fig. 4A). This lack of MCF-7-vector tumor formation in the absence of estrogen is consistent with previously published studies [36]. To further validate the mechanism of MEK5 mediated hormone independent tumorigenesis, tumors from the initial mouse study (Figure 1C) with estrogen treated animals were processed for H&E and IHC staining for ER-α and PgR expression (Fig. 4B). Results demonstrate a loss of ER-α expression along with a loss of estrogen-stimulated PgR expression in the MCF-7-MEK5 tumors compared to MCF-7-vector. MEK5 expressing tumors also displayed increased cellularity of H&E sections compared to MCF-7-vector tumors (Fig. 4B). These findings further suggest a role for MEK5 in progression to an ER-α (−) and hormone-independent phenotype.

### Knockdown of ERK5 Reverses Hormone Independence and Restores ER-α expression

**Figure 5:**
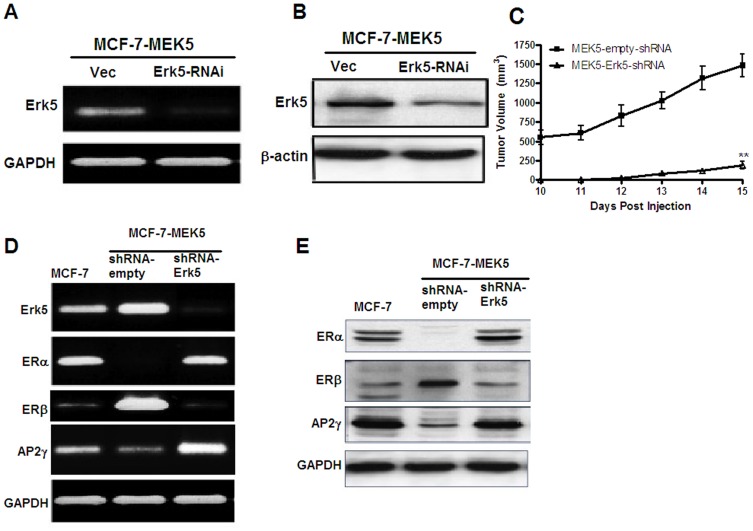
ERK5-RNA interference suppresses MCF-7-MEK5 tumor growth and restores ER-α protein expression. MCF-7-MEK5 cells were transfected with empty vector or ERK5 shRNA. Decreased expression of ERK5 was confirmed with (A) RT-PCR and (B) western blot analysis. (C) MCF-7-MEK5-(vector) or MCF-7-MEK5-(ERK5-shRNA) cells (5×10^5^) were injected into the mammary fat pad of Nu/Nu mice in the presence of E2 pellets (0.72 mg, 60 day release) (n = 10/group). Tumor growth was monitored daily after palpable tumor formation. Points represent mean tumor volume ± SEM; **, P<0.01. (D) RT-PCR analysis of ER-α, ER-β, AP2γ, ERK5 expression or GADPH (control) expression. Data shown is representative of analysis of three independent experiments. (E) Western blot analysis of ER-α, ER-β, AP2γ expression with GAPDH measured as control. Blots shown are representative of three independent experiments.

To confirm a role for the MEK5-ERK5 signaling pathway in the hormone independent phenotype of MEK5 overexpressing cells, shRNA ERK5 or vector constructs were stably transfected into MCF-7-MEK5 cells. Significant downregulation of ERK5 was observed in MCF-7-MEK5-ERK5-shRNA transfected cells compared to empty-shRNA vector (Fig. 5A and 5B). *In vivo* experiments further validated that ERK5 was essential for the increased tumor growth found in our previous *in vivo* experiments, as the ability of MEK5 expression to enhance tumor formation in immuno-compromised mice was suppressed by RNAi-mediated ablation of ERK5 (Fig. 5C).

We next used shRNA-targeted to ERK5 to investigate whether suppression of ERK5 signaling drives the loss of ER-α expression. MCF-7-vector, MCF-7-MEK5-(shRNA-empty) and MCF-7-MEK5-ERK5-shRNA cells were analyzed using RT-PCR and Western Blot for expression of ER-α. These data show that in the MEK5 overexpressing cells, ER-α is downregulated at both the mRNA (Fig. 5D) and protein (Fig. 5E) levels. Conversely, blockade of ERK5 by shRNA-ERK5 restored ER-α expression. Interestingly, ER-β expression and ER-α expression demonstrated an inverse relationship in MEK5 overexpressing cells and MEK5-ERK5-shRNA cells. Expression of ERK5 directly correlated with mRNA and protein expression of ER-β while shRNA knockdown of ERK5 inhibited expression of ER-β (Fig. 5D–E).

We next sought to investigate the mechanism of MEK5 mediated ER-α suppression. The above microarray findings revealed an inverse correlation between AP2 gamma (γ) and MEK5 expression, with MCF-7-MEK5 cells exhibiting markedly decreased AP2γ transcription compared to MCF-7-vector (Table 2). The transcription factor AP2γ regulates expression of ER and is known to increase expression ER-α and decrease expression of ER-β [45]–[50]. Therefore, we investigated whether there was an association between MEK5-ERK5 signaling pathway and AP2γ expression. As seen in Figure 5, we demonstrate an inverse correlation between ERK5 expression and the transcription factor AP2γ at both the mRNA (Fig. 5D) and protein (Fig. 5E) levels. This decrease in AP2γ expression in MCF-7-MEK5 cells is consistent with the above microarray analysis (Table 2). shRNA knockdown of ERK5 reversed the AP2γ suppression and restored ER-α expression in the MEK5 overexpressing cells (Figure 5D–E). Taken together, these results suggest that AP2γ may be involved in MEK5 mediated ER-α suppression.

To better understand the correlation between MEK5 induced repression of ER-α in the MCF-7 breast cancer cell line we next transiently overexpressed the AP2γ transcription factor in the MCF-7-MEK5 cell line and performed qRT-PCR to determine ERα expression levels. While a significant increase in expression of AP2γ was observed in MCF-7-MEK5 cells transiently transfected with AP2γ there was no observed increase in ER-α gene expression (Figure S4A) despite an increase in AP2γ (Figure S4B). This data suggests that while MEK5 may alter AP2γ gene expression, there is no significant correlation between AP2γ and ER-α expression in this cell line.

## Discussion

While recent evidence indicates a role for MAPK signaling in cancer oncogenesis and metastasis, the role of the MEK5-ERK5 pathway in breast cancer progression remains poorly understood. Currently, there is little data on the activation status of the MEK5-ERK5 pathway among breast cancer patients [13]. In this study we show clinical relevance of the MEK5-ERK5 signaling pathway through measurement of activated ERK5 in clinical tumor samples. We demonstrate activated ERK5 in 76.9% of breast biopsy samples as well as differences in pERK5 levels across breast cancer cell lines. Interestingly, ERK5 correlated with decreased ER-α protein expression in these breast cancer cells. Recent evidence suggests that MAPKs, namely ERK1/2, may regulate ER-α expression [8], [9], [10]. To our knowledge, this connection between ERK5 and ER-α expression has yet to be described in the literature.

**Figure 6:**
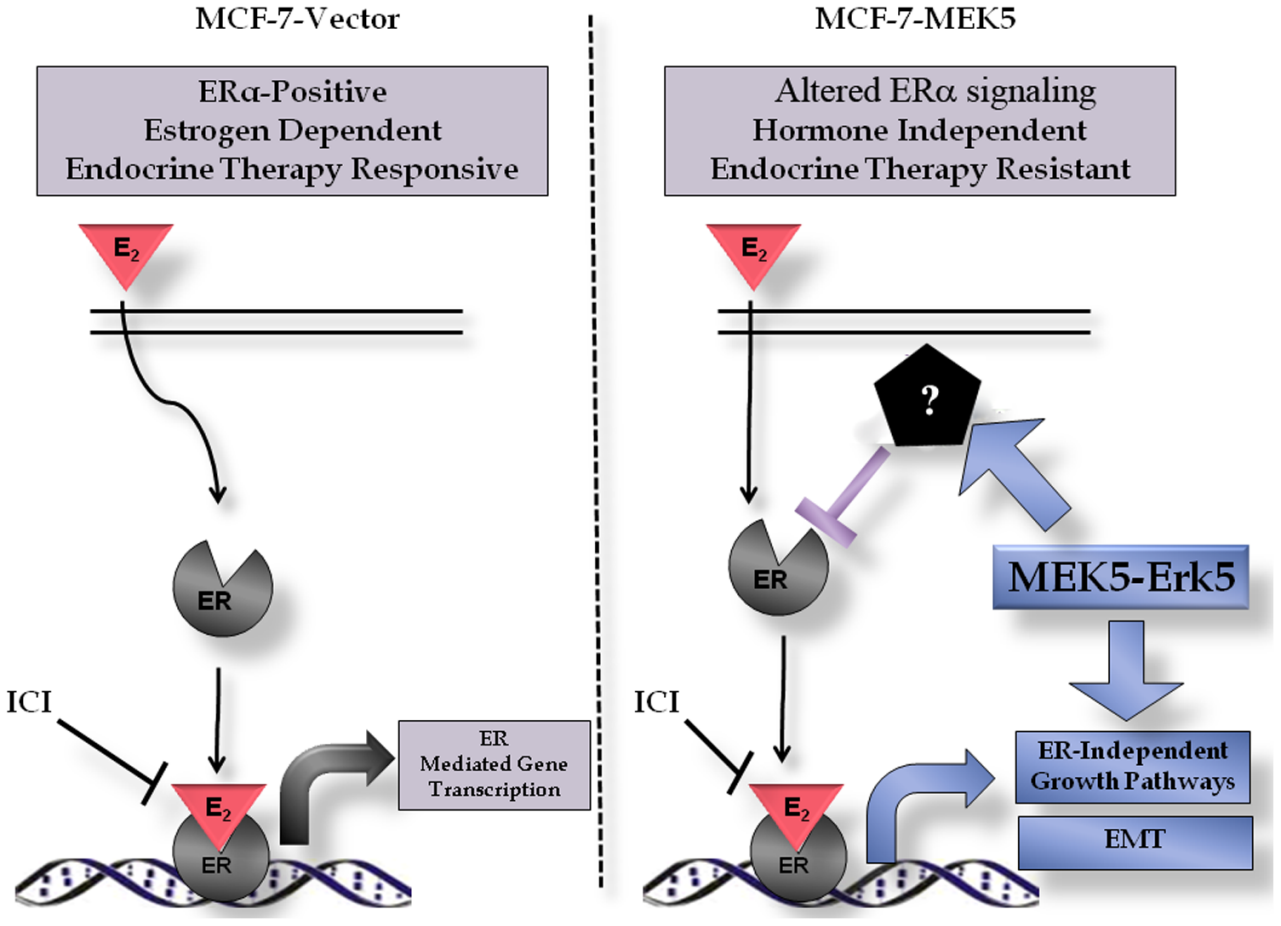
MEK5-mediated Hormone Independence. (A) MCF-7-vector (ERK5-negative) cells are estrogen-dependent. (B) MCF-7-MEK5 cells demonstrate hormone-independent cell proliferation and tumor growth.

We further elucidated the relationship between MEK5-ERK5 signaling and the ER regulated pathways. Expression of MEK5 suppressed ER-α, but not ER-β protein levels, and abrogated downstream ERE transcriptional activity and E2 induced gene transcription. ER-α expression in MCF-7-MEK5 cells could be restored using shRNA knockdown of ERK5. MEK5 mediated ER-α suppression conferred resistance to the endocrine therapies fulvestrant and tamoxifen *in vitro* and promoted hormone independent tumor growth *in vivo*. This is the first study to link MEK5-ERK5 signaling with ER expression and endocrine therapy resistance. As seen in Figure 6, we propose a model for MEK5/ERK5 signaling crosstalk with ER-α signaling to give rise to endocrine resistance and increased EMT. Through global gene expression profiling, we demonstrated MEK5 expression not only abrogated ER-α signaling, but also enhanced alternative mitogenic and survival signaling pathways to promote tumor growth. Pathway analysis of MEK5 cells revealed increased activation of ER independent growth signaling pathways, including MAPK, ERBB and PI3K-AKT, which may contribute to the hormone independent tumor growth of these cells.

There are several potential mechanisms for MEK5-induced repression of ER-α including regulation of downstream transcription factors. We found increased expression of the transcription factor TWIST1, which is known to decrease protein levels of ER-α in order to promote endocrine therapy resistance [51]. MEK5 cells also exhibited increased NF-κB mediated gene expression (data not shown), which is associated with the loss of ER-α and increased hormone independent survival [52]. The MEK5-ERK5 pathway is less understood than other MAPK pathways and the downstream targets of MEK5-ERK5 may not be fully identified.

Pathway analysis of our microarray data further revealed enhanced EMT-associated gene changes associated with MEK5. We and others have reported increased expression of MEK5-ERK5 in mesenchymal breast cancer cells, however, the mechanism of this correlation has yet to be determined [34]. Here, we identified several markers involved in MEK-induced EMT-associated gene changes, including increased expression of the ZEB family of zinc proteins and the TWIST1, SNAI1 and SNAI2 transcription factors. ZEB1/2 and SNAIs are known to alter E-cadherin mediated cellular adhesion whereas TWIST1 increases tumor seeding resulting in distant metastases [53], [54]. The EMT gene expression changes associated with MEK5 identified in this study corroborate previously published findings of MEK5 induced progression of breast cancer to a mesenchymal phenotype.

Taken together, the findings presented here provide new insight into the signaling mechanisms that regulation progression of breast cancer to hormone independence. Our data demonstrate a critical role for MEK5-ERK5 signaling in the progression to a hormone independent and EMT phenotype of breast carcinoma. Further studies are needed to better our understanding of the potential activators of MEK5-ERK5 and subsequent downstream signaling. However, our findings demonstrate the clinical relevance and therapeutic potential of targeting the MEK5-ERK5 pathway in the treatment of endocrine resistant breast cancer.

## Supporting Information

Figure S1 **Phosphorylated Erk5 in Human Ovarian Carcinoma.** Human breast ovarian tissue samples were collected and stained with anti-p-ERK5 (Thr218/Tyr220).
(TIF)

Figure S2 **Clustering Analyses of ER Target Gene Expression.** Red color indicates up-regulation and green color indicates down-regulation. Trees above are sample clusters.
(TIF)

Figure S3 **Clustering analyses of EMT Signature Gene Expression.** Red color indicates up-regulation and green color indicates down-regulation. Trees above are sample clusters.
(TIF)

Figure S4 **ER-α expression following overexpression of AP2γ in the MCF-7-MEK5 cell line.** Results represent q RT-PCR for (A) ER-α and (B) AP2γ in the MCF-7-MEK5 cell line transiently transfected with AP2γ or vector for 24 hours. Normalization was to beta-actin and MCF-7-MEK5-vector cells designated as 1. Cells were grown in 10% FBS DMEM.
(TIF)

Table S1 **Pathway analysis of MEK5 associated gene expression.**
(DOCX)

Table S2 **MEK5 induced EMT gene expression changes.**
(DOCX)

Table S3 **TNF pathway alterations associated with MEK5 expression.**
(DOCX)

Table S4 **Altered ER mediated gene expression in MEK5 expressing cells.**
(DOCX)
