# Supplementary figures and images for: MEK5/ERK5 Signaling Suppresses Estrogen Receptor Expression and Promotes Hormone-Independent Tumorigenesis
Source: PLoS One. 2013 Aug 9;8(8):e69291. doi: 10.1371/journal.pone.0069291 (PMC3739787; doi:10.1371/journal.pone.0069291)

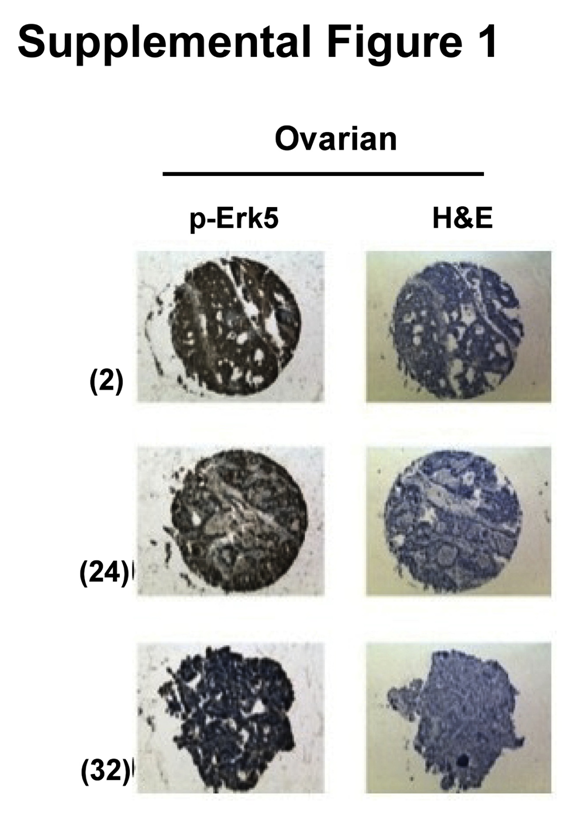

Supplement: Figure S1 — Phosphorylated Erk5 in Human Ovarian Carcinoma. Human breast ovarian tissue samples were collected and stained with anti-p-ERK5 (Thr218/Tyr220). (TIF) [file pone.0069291.s001.tif]

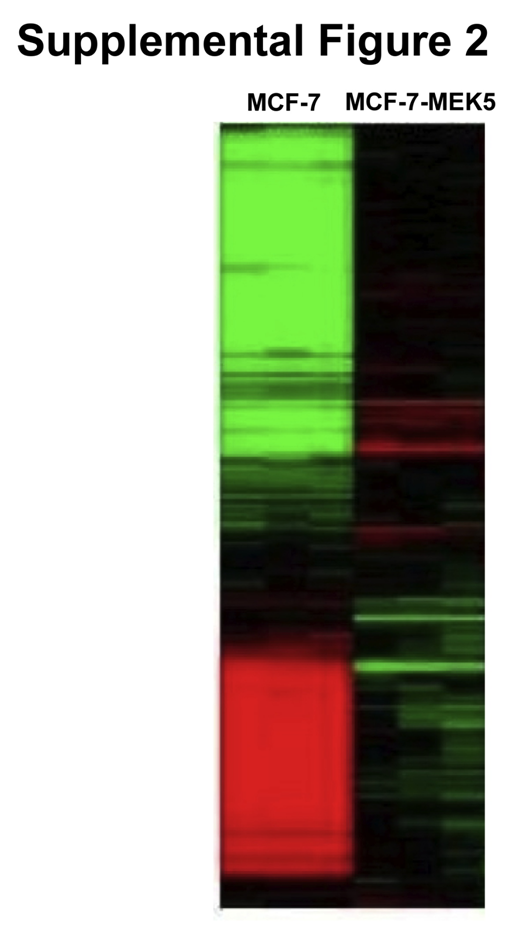

Supplement: Figure S2 — Clustering Analyses of ER Target Gene Expression. Red color indicates up-regulation and green color indicates down-regulation. Trees above are sample clusters. (TIF) [file pone.0069291.s002.tif]

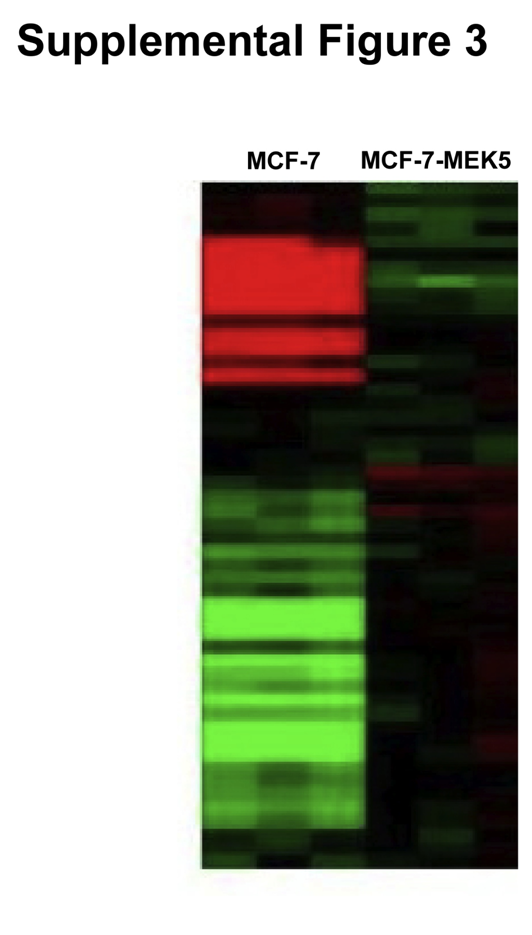

Supplement: Figure S3 — Clustering analyses of EMT Signature Gene Expression. Red color indicates up-regulation and green color indicates down-regulation. Trees above are sample clusters. (TIF) [file pone.0069291.s003.tif]

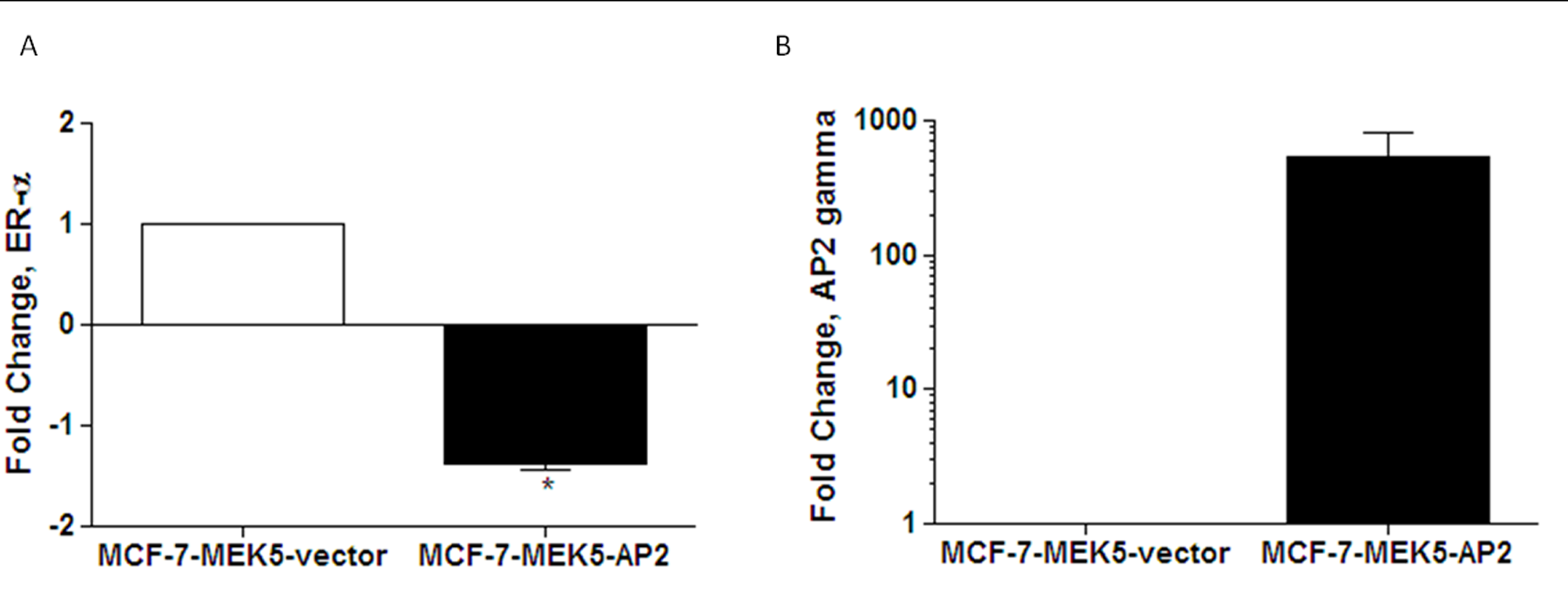

Supplement: Figure S4 — ER-α expression following overexpression of AP2γ in the MCF-7-MEK5 cell line. Results represent q RT-PCR for (A) ER-α and (B) AP2γ in the MCF-7-MEK5 cell line transiently transfected with AP2γ or vector for 24 hours. Normalization was to beta-actin and MCF-7-MEK5-vector cells designated as 1. Cells were grown in 10% FBS DMEM. (TIF) [file pone.0069291.s004.tif]
