# Supplementary material for: MEK5/ERK5 Signaling Suppresses Estrogen Receptor Expression and Promotes Hormone-Independent Tumorigenesis
Source: PLoS One. 2013 Aug 9;8(8):e69291. doi: 10.1371/journal.pone.0069291 (PMC3739787; doi:10.1371/journal.pone.0069291)
Supplement: Table S1 — Pathway analysis of MEK5 associated gene expression. (DOCX) [file pone.0069291.s005.docx]

**Supplemental Table 1. Pathway Analysis and Corresponding Gene Expression Changes Associated with MEK5 Expression**

| *Gene Symbol* | | *Fold Change (MEK5 vs. VEC)* | *Gene Name* | |  |  |
| --- | --- | --- | --- | --- | --- | --- |
|  |  |  |  |  | |  |
| Leukocyte Transendothelial Migration | | |  |  | |  |
|  | ACTN1 | -2.8211 | actinin, alpha 1 | | |  |
|  | ACTN2 | 2.5575 | actinin, alpha 2 | | |  |
|  | CD99 | 2.24442 | CD99 molecule | | |  |
|  | CLDN1 | -3.5734 | claudin 1 |  | |  |
|  | CLDN3 | -6.27181 | claudin 3 |  | |  |
|  | CLDN4 | -9.77984 | claudin 4 |  | |  |
|  | CLDN7 | -21.8632 | claudin 7 |  | |  |
|  | CXCL12 | -5.36674 | chemokine (C-X-C motif) ligand 12 (stromal cell-derived factor 1) | | | |
|  | F11R | -2.293 | F11 receptor |  | |  |
|  | GNAI1 | 35.2329 | guanine nucleotide binding protein (G protein), alpha inhibiting | | | |
|  | ITGA4 | 22.6432 | integrin, alpha 4 (antigen CD49D, alpha 4 subunit of VLA-4 receptor) | | | |
|  | JAM2 | 4.8544 | junctional adhesion molecule 2 | | | |
|  | JAM3 | 15.2062 | junctional adhesion molecule 3 | | | |
|  | MLLT4 | 3.76221 | myeloid/lymphoid or mixed-lineage leukemia (trithorax homolog, Drosophila); translocated to, 4 | | | |
|  | MMP2 | 4.86963 | matrix metallopeptidase 2 (gelatinase A, 72kDa gelatinase, 72kDa | | | |
|  | MSN | 21.1665 | moesin |  | |  |
|  | PIK3CB | -2.34109 | phosphoinositide-3-kinase, catalytic, beta polypeptide | | | |
|  | PIK3R1 | -2.03668 | phosphoinositide-3-kinase, regulatory subunit 1 (alpha) | | | |
|  | PLCG1 | -2.56212 | phospholipase C, gamma 1 (EC:3.1.4.11) | | | |
|  | PRKCA | 2.00421 | protein kinase C, alpha (EC:2.7.11.13) | | | |
|  | PRKCB | 2.17984 | protein kinase C, beta (EC:2.7.11.13) | | | |
|  | PXN | -2.69475 | paxillin |  | |  |
|  | RASSF5 | 2.51783 | Ras association (RalGDS/AF-6) domain family member 5 | | | |
|  | VAV3 | -3.18735 | vav 3 guanine nucleotide exchange factor | | | |
|  |  |  |  |  | |  |
|  |  |  |  |  | |  |
| Cell adhesion molecules (CAMs) | | |  |  | |  |
|  | CD58 | -2.14035 | CD58 molecule | | |  |
|  | CD99 | 2.24442 | CD99 molecule | | |  |
|  | CDH1 | -46.3752 | cadherin 1, type 1, E-cadherin (epithelial) | | | |
|  | CDH2 | 48.2462 | cadherin 2, type 1, N-cadherin (neuronal) | | | |
|  | CDH3 | -6.16036 | cadherin 3, type 1, P-cadherin (placental) | | | |
|  | CLDN1 | -3.5734 | claudin 1 |  | |  |
|  | CLDN3 | -6.27181 | claudin 3 |  | |  |
|  | CLDN4 | -9.77984 | claudin 4 |  | |  |
|  | CLDN7 | -21.8632 | claudin 7 |  | |  |
|  | CNTN1 | 25.8007 | contactin 1 |  | |  |
|  | F11R | -2.293 | F11 receptor |  | |  |
|  | HLA-DPA1 | 20.1137 | major histocompatibility complex, class II, DP alpha 1 | | | |
|  | HLA-DPB1 | 5.86684 | major histocompatibility complex, class II, DP beta 1 | | | |
|  | ICAM3 | -2.37075 | intercellular adhesion molecule 3 | | | |
|  | ICOSLG | -2.27659 | inducible T-cell co-stimulator ligand | | | |
|  | ITGA4 | 22.6432 | integrin, alpha 4 (antigen CD49D, alpha 4 subunit of VLA-4 receptor) | | | |
|  | ITGA6 | 3.60386 | integrin, alpha 6 | | |  |
|  | ITGA8 | 15.1314 | integrin, alpha 8 | | |  |
|  | JAM2 | 4.8544 | junctional adhesion molecule 2 | | | |
|  | JAM3 | 15.2062 | junctional adhesion molecule 3 | | | |
|  | L1CAM | -3.58467 | L1 cell adhesion molecule | | | |
|  | NCAM1 | 10.4543 | neural cell adhesion molecule 1 | | | |
|  | NCAM2 | -5.78961 | neural cell adhesion molecule 2 | | | |
|  | NEO1 | 2.05386 | neogenin homolog 1 (chicken) | | | |
|  | NLGN1 | 15.2477 | neuroligin 1 |  | |  |
|  | NRCAM | -2.33665 | neuronal cell adhesion molecule | | | |
|  | PTPRM | 9.03533 | protein tyrosine phosphatase, receptor type, M (EC:3.1.3.48) | | | |
|  | PVRL1 | -2.06133 | poliovirus receptor-related 1 (herpesvirus entry mediator C) | | | |
|  | PVRL3 | 18.2954 | poliovirus receptor-related 3 | | | |
|  | SDC1 | -3.29802 | syndecan 1 |  | |  |
|  | SDC4 | -4.72484 | syndecan 4 |  | |  |
|  | VCAN | 54.8565 | versican |  | |  |
|  |  |  |  |  | |  |
| Adherens junction | |  |  |  | |  |
|  | ACTN1 | -2.8211 | actinin, alpha 1 | | |  |
|  | ACTN2 | 2.5575 | actinin, alpha 2 | | |  |
|  | BAIAP2 | -2.13381 | BAI1-associated protein 2 | | | |
|  | CDH1 | -46.3752 | cadherin 1, type 1, E-cadherin (epithelial) | | | |
|  | FER | 2.1969 | fer (fps/fes related) tyrosine kinase (EC:2.7.10.2) | | | |
|  | FGFR1 | 2.06284 | fibroblast growth factor receptor 1 (EC:2.7.10.1) | | | |
|  | FYN | 15.4244 | FYN oncogene related to SRC, FGR, YES (EC:2.7.10.2) | | | |
|  | LEF1 | 8.68433 | lymphoid enhancer-binding factor 1 | | | |
|  | MET | 2.94204 | met proto-oncogene (hepatocyte growth factor receptor) (EC:2.7.10.1) | | | |
|  | MLLT4 | 3.76221 | myeloid/lymphoid or mixed-lineage leukemia (trithorax homolog, Drosophila); translocated to, 4 | | | |
|  | PTPN6 | -5.87281 | protein tyrosine phosphatase, non-receptor type 6 (EC:3.1.3.48) | | | |
|  | PTPRM | 9.03533 | protein tyrosine phosphatase, receptor type, M (EC:3.1.3.48) | | | |
|  | PVRL1 | -2.06133 | poliovirus receptor-related 1 (herpesvirus entry mediator C) | | | |
|  | PVRL3 | 18.2954 | poliovirus receptor-related 3 | | | |
|  | RAC3 | 2.73518 | ras-related C3 botulinum toxin substrate 3 (rho family, small GTP | | | |
|  | SMAD2 | 2.13231 | SMAD family member 2 | | | |
|  | SMAD3 | -2.58977 | SMAD family member 3 | | | |
|  | SMAD4 | 2.76934 | SMAD family member 4 | | | |
|  | SNAI2 | 3.79477 | snail homolog 2 (Drosophila) | | | |
|  | SORBS1 | 2.09012 | sorbin and SH3 domain containing 1 | | | |
|  | SSX2IP | 2.10417 | synovial sarcoma, X breakpoint 2 interacting protein | | | |
|  | TCF7 | 2.28385 | transcription factor 7 (T-cell specific, HMG-box) | | | |
|  | WASF1 | 4.43784 | WAS protein family, member 1 | | | |
|  |  |  |  |  | |  |
| Axon guidance | |  |  |  | |  |
|  | CFL2 | 3.63673 | cofilin 2 (muscle) | | |  |
|  | CXCL12 | -5.36674 | chemokine (C-X-C motif) ligand 12 (stromal cell-derived factor 1) | | | |
|  | DPYSL2 | 2.16564 | dihydropyrimidinase-like 2 | | | |
|  | DPYSL5 | 3.28194 | dihydropyrimidinase-like 5 | | | |
|  | EFNA1 | -3.61907 | ephrin-A1 |  | |  |
|  | EFNA3 | -2.01449 | ephrin-A3 |  | |  |
|  | EFNA4 | -3.3682 | ephrin-A4 |  | |  |
|  | EFNA5 | 3.48899 | ephrin-A5 |  | |  |
|  | EFNB2 | 9.34578 | ephrin-B2 |  | |  |
|  | EPHA1 | -2.85231 | EPH receptor A1 (EC:2.7.10.1) | | | |
|  | EPHA3 | 12.8251 | EPH receptor A3 (EC:2.7.10.1) | | | |
|  | EPHA5 | 5.2678 | EPH receptor A5 (EC:2.7.10.1) | | | |
|  | EPHA6 | 5.97295 | EPH receptor A6 (EC:2.7.10.1) | | | |
|  | EPHA7 | 61.3695 | EPH receptor A7 (EC:2.7.10.1) | | | |
|  | FYN | 15.4244 | FYN oncogene related to SRC, FGR, YES (EC:2.7.10.2) | | | |
|  | GNAI1 | 35.2329 | guanine nucleotide binding protein (G protein), alpha inhibiting | | | |
|  | L1CAM | -3.58467 | L1 cell adhesion molecule | | | |
|  | MET | 2.94204 | met proto-oncogene (hepatocyte growth factor receptor) (EC:2.7.10.1) | | | |
|  | NFATC2 | -12.9791 | nuclear factor of activated T-cells, cytoplasmic, calcineurin-dependent 2 | | | |
|  | NRAS | -2.4873 | neuroblastoma RAS viral (v-ras) oncogene homolog | | | |
|  | NRP1 | -2.78481 | neuropilin 1 |  | |  |
|  | PAK1 | 5.15964 | p21 protein (Cdc42/Rac)-activated kinase 1 (EC:2.7.11.1) | | | |
|  | PLXNA2 | 2.07389 | plexin A2 |  | |  |
|  | PLXNC1 | 2.6874 | plexin C1 |  | |  |
|  | RAC3 | 2.73518 | ras-related C3 botulinum toxin substrate 3 (rho family, small GTP | | | |
|  | RHOD | -9.53758 | ras homolog gene family, member D | | | |
|  | RND1 | -3.36874 | Rho family GTPase 1 | | |  |
|  | ROBO1 | 5.04723 | roundabout, axon guidance receptor, homolog 1 (Drosophila) | | | |
|  | ROBO2 | 8.53883 | roundabout, axon guidance receptor, homolog 2 (Drosophila) | | | |
|  | SEMA3A | 10.746 | sema domain, immunoglobulin domain (Ig), short basic domain, secreted, (semaphorin) 3A | | | |
|  | SEMA3B | -4.26187 | sema domain, immunoglobulin domain (Ig), short basic domain, secreted, (semaphorin) 3B | | | |
|  | SEMA3C | -5.18998 | sema domain, immunoglobulin domain (Ig), short basic domain, secreted, (semaphorin) 3C | | | |
|  | SEMA3D | 15.0724 | sema domain, immunoglobulin domain (Ig), short basic domain, secreted, (semaphorin) 3D | | | |
|  | SEMA3F | -2.19116 | sema domain, immunoglobulin domain (Ig), short basic domain, secreted, (semaphorin) 3F | | | |
|  | SEMA4A | -2.60913 | sema domain, immunoglobulin domain (Ig), transmembrane domain (TM) | | | |
|  | SEMA4C | -2.62932 | sema domain, immunoglobulin domain (Ig), transmembrane domain (TM) | | | |
|  | SEMA5A | -2.95828 | sema domain, seven thrombospondin repeats (type 1 and type 1-like), transmembrane domain (TM) and short cytoplasmic domain, (semaphorin) | | | |
|  | SEMA6A | 8.10394 | sema domain, transmembrane domain (TM), and cytoplasmic domain, (semaphorin) 6A | | | |
|  | SEMA6D | 3.08378 | sema domain, transmembrane domain (TM), and cytoplasmic domain, (semaphorin) 6D | | | |
|  | SLIT2 | 85.9199 | slit homolog 2 (Drosophila) | | | |
|  | SRGAP1 | -5.20779 | SLIT-ROBO Rho GTPase activating protein 1 | | | |
|  | UNC5B | 2.31067 | unc-5 homolog B (C. elegans) | | | |
|  | UNC5C | 7.05763 | unc-5 homolog C (C. elegans) | | | |
|  |  |  |  |  | |  |
| Pathways in cancer | |  |  |  | |  |
|  | AKT1 | -2.75982 | v-akt murine thymoma viral oncogene homolog 1 (EC:2.7.11.1) | | | |
|  | AKT3 | 86.282 | v-akt murine thymoma viral oncogene homolog 3 (protein kinase B, gamma) (EC:2.7.11.1) | | | |
|  | AR | -10.9909 | androgen receptor | | |  |
|  | BCL2 | -3.16498 | B-cell CLL/lymphoma 2 | | | |
|  | BCL2L1 | -3.13264 | BCL2-like 1 |  | |  |
|  | CASP3 | 3.41838 | caspase 3, apoptosis-related cysteine peptidase (EC:3.4.22.56) | | | |
|  | CASP8 | -2.04954 | caspase 8, apoptosis-related cysteine peptidase (EC:3.4.22.61) | | | |
|  | CCND1 | -7.59907 | cyclin D1 |  | |  |
|  | CDH1 | -46.3752 | cadherin 1, type 1, E-cadherin (epithelial) | | | |
|  | CDK6 | 6.74438 | cyclin-dependent kinase 6 (EC:2.7.11.22) | | | |
|  | CDKN2A | 10.6198 | cyclin-dependent kinase inhibitor 2A (melanoma, p16, inhibits CDK4) | | | |
|  | COL4A1 | 4.24975 | collagen, type IV, alpha 1 | | | |
|  | COL4A2 | 4.49578 | collagen, type IV, alpha 2 | | | |
|  | DAPK1 | 14.7954 | death-associated protein kinase 1 (EC:2.7.11.1) | | | |
|  | EPAS1 | -2.18472 | endothelial PAS domain protein 1 | | | |
|  | ETS1 | 3.502 | v-ets erythroblastosis virus E26 oncogene homolog 1 (avian) | | | |
|  | EVI1 | 5.27319 | ecotropic viral integration site 1 | | | |
|  | FAS | 2.23839 | Fas (TNF receptor superfamily, member 6) | | | |
|  | FGF13 | 3.43852 | fibroblast growth factor 13 | | | |
|  | FGF2 | 3.23962 | fibroblast growth factor 2 (basic) | | | |
|  | FGF5 | 2.26717 | fibroblast growth factor 5 | | | |
|  | FGF9 | 7.08775 | fibroblast growth factor 9 (glia-activating factor) | | | |
|  | FGFR1 | 2.06284 | fibroblast growth factor receptor 1 (EC:2.7.10.1) | | | |
|  | FGFR2 | 2.69989 | fibroblast growth factor receptor 2 (EC:2.7.10.1) | | | |
|  | FN1 | -2.61255 | fibronectin 1 |  | |  |
|  | FOS | -12.6327 | v-fos FBJ murine osteosarcoma viral oncogene homolog | | | |
|  | FZD6 | -2.72208 | frizzled homolog 6 (Drosophila) | | | |
|  | GLI3 | 2.07366 | GLI family zinc finger 3 | | |  |
|  | GSTP1 | 44.5587 | glutathione S-transferase pi 1 (EC:2.5.1.18) | | | |
|  | HHIP | 2.18772 | hedgehog interacting protein | | | |
|  | IL6 | -2.01775 | interleukin 6 (interferon, beta 2) | | | |
|  | IL8 | -3.05642 | interleukin 8 |  | |  |
|  | ITGA2 | -4.45339 | integrin, alpha 2 (CD49B, alpha 2 subunit of VLA-2 receptor) | | | |
|  | ITGA3 | -4.33893 | integrin, alpha 3 (antigen CD49C, alpha 3 subunit of VLA-3 receptor) | | | |
|  | ITGA6 | 3.60386 | integrin, alpha 6 | | |  |
|  | JUP | -3.42739 | junction plakoglobin | | |  |
|  | KIT | 17.1907 | v-kit Hardy-Zuckerman 4 feline sarcoma viral oncogene homolog | | | |
|  | LAMA1 | 3.89938 | laminin, alpha 1 | | |  |
|  | LAMA3 | 2.06781 | laminin, alpha 3 | | |  |
|  | LAMA4 | 26.1891 | laminin, alpha 4 | | |  |
|  | LAMB1 | 5.53191 | laminin, beta 1 | | |  |
|  | LAMB3 | -2.20262 | laminin, beta 3 | | |  |
|  | LAMC2 | -3.54862 | laminin, gamma 2 | | |  |
|  | LEF1 | 8.68433 | lymphoid enhancer-binding factor 1 | | | |
|  | MAPK8 | 2.03382 | mitogen-activated protein kinase 8 (EC:2.7.11.24) | | | |
|  | MET | 2.94204 | met proto-oncogene (hepatocyte growth factor receptor) (EC:2.7.10.1) | | | |
|  | MMP1 | -2.86873 | matrix metallopeptidase 1 (interstitial collagenase) (EC:3.4.24.7) | | | |
|  | MMP2 | 4.86963 | matrix metallopeptidase 2 (gelatinase A, 72kDa gelatinase, 72kDa | | | |
|  | NFKB2 | -2.08869 | nuclear factor of kappa light polypeptide gene enhancer in B-cells 2 | | | |
|  | NFKBIA | -3.54182 | nuclear factor of kappa light polypeptide gene enhancer in B-cells | | | |
|  | NRAS | -2.4873 | neuroblastoma RAS viral (v-ras) oncogene homolog | | | |
|  | PDGFA | -2.22123 | platelet-derived growth factor alpha polypeptide | | | |
|  | PDGFRA | 2.27842 | platelet-derived growth factor receptor, alpha polypeptide | | | |
|  | PGF | 2.69592 | placental growth factor | | |  |
|  | PIAS3 | -2.87942 | protein inhibitor of activated STAT, 3 | | | |
|  | PIK3CB | -2.34109 | phosphoinositide-3-kinase, catalytic, beta polypeptide | | | |
|  | PIK3R1 | -2.03668 | phosphoinositide-3-kinase, regulatory subunit 1 (alpha) | | | |
|  | PLCG1 | -2.56212 | phospholipase C, gamma 1 (EC:3.1.4.11) | | | |
|  | PLD1 | 2.19983 | phospholipase D1, phosphatidylcholine-specific (EC:3.1.4.4) | | | |
|  | PRKCA | 2.00421 | protein kinase C, alpha (EC:2.7.11.13) | | | |
|  | PRKCB | 2.17984 | protein kinase C, beta (EC:2.7.11.13) | | | |
|  | PTCH1 | 2.19434 | patched homolog 1 (Drosophila) | | | |
|  | RAC3 | 2.73518 | ras-related C3 botulinum toxin substrate 3 (rho family, small GTP | | | |
|  | RARA | -2.07914 | retinoic acid receptor, alpha | | | |
|  | RARB | 8.01838 | retinoic acid receptor, beta | | | |
|  | RASSF5 | 2.51783 | Ras association (RalGDS/AF-6) domain family member 5 | | | |
|  | RET | -5.52908 | ret proto-oncogene (EC:2.7.10.1) | | | |
|  | RUNX1 | -2.13455 | runt-related transcription factor 1 | | | |
|  | RUNX1T1 | 3.6035 | runt-related transcription factor 1; translocated to, 1 (cyclin | | | |
|  | SKP2 | 5.15539 | S-phase kinase-associated protein 2 (p45) | | | |
|  | SMAD2 | 2.13231 | SMAD family member 2 | | | |
|  | SMAD3 | -2.58977 | SMAD family member 3 | | | |
|  | SMAD4 | 2.76934 | SMAD family member 4 | | | |
|  | SMO | 2.43504 | smoothened homolog (Drosophila) | | | |
|  | STAT1 | -3.20436 | signal transducer and activator of transcription 1, 91kDa | | | |
|  | TCF7 | 2.28385 | transcription factor 7 (T-cell specific, HMG-box) | | | |
|  | TGFA | -4.90783 | transforming growth factor, alpha | | | |
|  | TGFB1 | -2.2187 | transforming growth factor, beta 1 | | | |
|  | TGFB2 | -4.55364 | transforming growth factor, beta 2 | | | |
|  | WNT11 | 3.22419 | wingless-type MMTV integration site family, member 11 | | | |
|  | WNT5A | 5.96226 | wingless-type MMTV integration site family, member 5A | | | |
|  |  |  |  |  | |  |
| Focal Adhesion | |  |  |  | |  |
|  | ACTN1 | -2.8211 | actinin, alpha 1 | | |  |
|  | ACTN2 | 2.5575 | actinin, alpha 2 | | |  |
|  | AKT1 | -2.75982 | v-akt murine thymoma viral oncogene homolog 1 (EC:2.7.11.1) | | | |
|  | AKT3 | 86.282 | v-akt murine thymoma viral oncogene homolog 3 (protein kinase B, gamma) (EC:2.7.11.1) | | | |
|  | BCL2 | -3.16498 | B-cell CLL/lymphoma 2 | | | |
|  | CAPN2 | 2.17305 | calpain 2, (m/II) large subunit (EC:3.4.22.53) | | | |
|  | CCND1 | -7.59907 | cyclin D1 |  | |  |
|  | CCND2 | 30.8703 | cyclin D2 |  | |  |
|  | COL2A1 | 4.7679 | collagen, type II, alpha 1 | | | |
|  | COL4A1 | 4.24975 | collagen, type IV, alpha 1 | | | |
|  | COL4A2 | 4.49578 | collagen, type IV, alpha 2 | | | |
|  | COL6A1 | 2.4514 | collagen, type VI, alpha 1 | | | |
|  | FLNC | 2.93542 | filamin C, gamma (actin binding protein 280) | | | |
|  | FLT1 | 7.28125 | fms-related tyrosine kinase 1 (vascular endothelial growth | | | |
|  | FN1 | -2.61255 | fibronectin 1 |  | |  |
|  | FYN | 15.4244 | FYN oncogene related to SRC, FGR, YES (EC:2.7.10.2) | | | |
|  | ITGA2 | -4.45339 | integrin, alpha 2 (CD49B, alpha 2 subunit of VLA-2 receptor) | | | |
|  | ITGA3 | -4.33893 | integrin, alpha 3 (antigen CD49C, alpha 3 subunit of VLA-3 receptor) | | | |
|  | ITGA4 | 22.6432 | integrin, alpha 4 (antigen CD49D, alpha 4 subunit of VLA-4 receptor) | | | |
|  | ITGA6 | 3.60386 | integrin, alpha 6 | | |  |
|  | ITGA8 | 15.1314 | integrin, alpha 8 | | |  |
|  | ITGB4 | -4.59781 | integrin, beta 4 | | |  |
|  | ITGB5 | -3.89526 | integrin, beta 5 | | |  |
|  | ITGB6 | -27.4734 | integrin, beta 6 | | |  |
|  | LAMA1 | 3.89938 | laminin, alpha 1 | | |  |
|  | LAMA3 | 2.06781 | laminin, alpha 3 | | |  |
|  | LAMA4 | 26.1891 | laminin, alpha 4 | | |  |
|  | LAMB1 | 5.53191 | laminin, beta 1 | | |  |
|  | LAMB3 | -2.20262 | laminin, beta 3 | | |  |
|  | LAMC2 | -3.54862 | laminin, gamma 2 | | |  |
|  | MAPK8 | 2.03382 | mitogen-activated protein kinase 8 (EC:2.7.11.24) | | | |
|  | MET | 2.94204 | met proto-oncogene (hepatocyte growth factor receptor) (EC:2.7.10.1) | | | |
|  | MYLK | -3.96761 | myosin light chain kinase (EC:2.7.11.18) | | | |
|  | PAK1 | 5.15964 | p21 protein (Cdc42/Rac)-activated kinase 1 (EC:2.7.11.1) | | | |
|  | PDGFA | -2.22123 | platelet-derived growth factor alpha polypeptide | | | |
|  | PDGFC | 11.677 | platelet derived growth factor C | | | |
|  | PDGFD | 3.45827 | platelet derived growth factor D | | | |
|  | PDGFRA | 2.27842 | platelet-derived growth factor receptor, alpha polypeptide | | | |
|  | PDPK1 | -2.00895 | 3-phosphoinositide dependent protein kinase-1 (EC:2.7.11.1) | | | |
|  | PGF | 2.69592 | placental growth factor | | |  |
|  | PIK3CB | -2.34109 | phosphoinositide-3-kinase, catalytic, beta polypeptide | | | |
|  | PIK3R1 | -2.03668 | phosphoinositide-3-kinase, regulatory subunit 1 (alpha) | | | |
|  | PRKCA | 2.00421 | protein kinase C, alpha (EC:2.7.11.13) | | | |
|  | PRKCB | 2.17984 | protein kinase C, beta (EC:2.7.11.13) | | | |
|  | PXN | -2.69475 | paxillin |  | |  |
|  | RAC3 | 2.73518 | ras-related C3 botulinum toxin substrate 3 (rho family, small GTP | | | |
|  | SHC4 | -3.80376 | SHC (Src homology 2 domain containing) family, member 4 | | | |
|  | THBS1 | -45.0525 | thrombospondin 1 | | |  |
|  | TNC | 2.28918 | tenascin C |  | |  |
|  | VAV3 | -3.18735 | vav 3 guanine nucleotide exchange factor | | | |
|  | ZYX | -2.19944 | zyxin |  | |  |
|  |  |  |  |  | |  |
| MAPK signaling pathway | | |  |  | |  |
|  | AKT1 | -2.75982 | v-akt murine thymoma viral oncogene homolog 1 (EC:2.7.11.1) | | | |
|  | AKT3 | 86.282 | v-akt murine thymoma viral oncogene homolog 3 (protein kinase B, gamma) (EC:2.7.11.1) | | | |
|  | ARRB1 | -2.54009 | arrestin, beta 1 | | |  |
|  | CACNA1D | -5.87193 | calcium channel, voltage-dependent, L type, alpha 1D subunit | | | |
|  | CACNA1H | -2.04841 | calcium channel, voltage-dependent, T type, alpha 1H subunit | | | |
|  | CACNA2D1 | 28.0104 | calcium channel, voltage-dependent, alpha 2/delta subunit 1 | | | |
|  | CACNA2D3 | 9.16775 | calcium channel, voltage-dependent, alpha 2/delta subunit 3 | | | |
|  | CACNB2 | 4.22789 | calcium channel, voltage-dependent, beta 2 subunit | | | |
|  | CACNB3 | -2.3007 | calcium channel, voltage-dependent, beta 3 subunit | | | |
|  | CACNB4 | 3.03472 | calcium channel, voltage-dependent, beta 4 subunit | | | |
|  | CACNG4 | -8.67682 | calcium channel, voltage-dependent, gamma subunit 4 | | | |
|  | CASP3 | 3.41838 | caspase 3, apoptosis-related cysteine peptidase (EC:3.4.22.56) | | | |
|  | CDC25B | -2.44609 | cell division cycle 25 homolog B (S. pombe) (EC:3.1.3.48) | | | |
|  | DDIT3 | -2.69424 | DNA-damage-inducible transcript 3 | | | |
|  | DUSP4 | -5.55732 | dual specificity phosphatase 4 (EC:3.1.3.16 3.1.3.48) | | | |
|  | ELK4 | -2.32092 | ELK4, ETS-domain protein (SRF accessory protein 1) | | | |
|  | EVI1 | 5.27319 | ecotropic viral integration site 1 | | | |
|  | FAS | 2.23839 | Fas (TNF receptor superfamily, member 6) | | | |
|  | FGF13 | 3.43852 | fibroblast growth factor 13 | | | |
|  | FGF2 | 3.23962 | fibroblast growth factor 2 (basic) | | | |
|  | FGF5 | 2.26717 | fibroblast growth factor 5 | | | |
|  | FGF9 | 7.08775 | fibroblast growth factor 9 (glia-activating factor) | | | |
|  | FGFR1 | 2.06284 | fibroblast growth factor receptor 1 (EC:2.7.10.1) | | | |
|  | FGFR2 | 2.69989 | fibroblast growth factor receptor 2 (EC:2.7.10.1) | | | |
|  | FLNC | 2.93542 | filamin C, gamma (actin binding protein 280) | | | |
|  | FOS | -12.6327 | v-fos FBJ murine osteosarcoma viral oncogene homolog | | | |
|  | GNG12 | 2.24456 | guanine nucleotide binding protein (G protein), gamma 12 | | | |
|  | HSPA1B | 2.18867 | heat shock 70kDa protein 1B | | | |
|  | HSPA1L | 2.15594 | heat shock 70kDa protein 1-like | | | |
|  | HSPA6 | -2.59467 | heat shock 70kDa protein 6 (HSP70B'') | | | |
|  | HSPB1 | -6.39327 | heat shock 27kDa protein 1 | | | |
|  | MAP2K5 | 2.55893 | mitogen-activated protein kinase kinase 5 (EC:2.7.12.2) | | | |
|  | MAP2K6 | 4.73239 | mitogen-activated protein kinase kinase 6 (EC:2.7.12.2) | | | |
|  | MAP3K11 | -2.27612 | mitogen-activated protein kinase kinase kinase 11 (EC:2.7.11.25) | | | |
|  | MAP3K14 | -2.61877 | mitogen-activated protein kinase kinase kinase 14 (EC:2.7.11.25) | | | |
|  | MAP3K4 | 2.95496 | mitogen-activated protein kinase kinase kinase 4 (EC:2.7.11.25) | | | |
|  | MAP4K3 | -2.50366 | mitogen-activated protein kinase kinase kinase kinase 3 | | | |
|  | MAPK8 | 2.03382 | mitogen-activated protein kinase 8 (EC:2.7.11.24) | | | |
|  | MAPKAPK2 | -3.37289 | mitogen-activated protein kinase-activated protein kinase 2 | | | |
|  | MAPT | -2.27302 | microtubule-associated protein tau | | | |
|  | MEF2C | 21.7039 | myocyte enhancer factor 2C | | | |
|  | MRAS | 2.8653 | muscle RAS oncogene homolog | | | |
|  | NFATC2 | -12.9791 | nuclear factor of activated T-cells, cytoplasmic, calcineurin-dependent 2 | | | |
|  | NFKB2 | -2.08869 | nuclear factor of kappa light polypeptide gene enhancer in B-cells 2 | | | |
|  | NRAS | -2.4873 | neuroblastoma RAS viral (v-ras) oncogene homolog | | | |
|  | PAK1 | 5.15964 | p21 protein (Cdc42/Rac)-activated kinase 1 (EC:2.7.11.1) | | | |
|  | PDGFA | -2.22123 | platelet-derived growth factor alpha polypeptide | | | |
|  | PDGFRA | 2.27842 | platelet-derived growth factor receptor, alpha polypeptide | | | |
|  | PLA2G10 | -2.69651 | phospholipase A2, group X (EC:3.1.1.4) | | | |
|  | PLA2G4A | 14.5133 | phospholipase A2, group IVA (cytosolic, calcium-dependent) | | | |
|  | PRKACB | 2.65663 | protein kinase, cAMP-dependent, catalytic, beta (EC:2.7.11.11) | | | |
|  | PRKCA | 2.00421 | protein kinase C, alpha (EC:2.7.11.13) | | | |
|  | PRKCB | 2.17984 | protein kinase C, beta (EC:2.7.11.13) | | | |
|  | RAC3 | 2.73518 | ras-related C3 botulinum toxin substrate 3 (rho family, small GTP | | | |
|  | RASGRF2 | 7.9654 | Ras protein-specific guanine nucleotide-releasing factor 2 | | | |
|  | RASGRP1 | -2.20114 | RAS guanyl releasing protein 1 (calcium and DAG-regulated) | | | |
|  | RELB | -2.04297 | v-rel reticuloendotheliosis viral oncogene homolog B | | | |
|  | RPS6KA5 | 2.12281 | ribosomal protein S6 kinase, 90kDa, polypeptide 5 (EC:2.7.11.1) | | | |
|  | RPS6KA6 | 4.21396 | ribosomal protein S6 kinase, 90kDa, polypeptide 6 (EC:2.7.11.1) | | | |
|  | STMN1 | 2.23532 | stathmin 1/oncoprotein 18 | | | |
|  | TGFB1 | -2.2187 | transforming growth factor, beta 1 | | | |
|  | TGFB2 | -4.55364 | transforming growth factor, beta 2 | | | |
|  | TNFRSF1A | -2.06155 | tumor necrosis factor receptor superfamily, member 1A | | | |
|  |  |  |  |  | |  |
| ECM-receptor interaction | | |  |  | |  |
|  | CD36 | -9.46501 | CD36 molecule (thrombospondin receptor) | | | |
|  | CD44 | -3.48838 | CD44 molecule (Indian blood group) | | | |
|  | COL2A1 | 4.7679 | collagen, type II, alpha 1 | | | |
|  | COL4A1 | 4.24975 | collagen, type IV, alpha 1 | | | |
|  | COL4A2 | 4.49578 | collagen, type IV, alpha 2 | | | |
|  | COL6A1 | 2.4514 | collagen, type VI, alpha 1 | | | |
|  | FN1 | -2.61255 | fibronectin 1 |  | |  |
|  | ITGA2 | -4.45339 | integrin, alpha 2 (CD49B, alpha 2 subunit of VLA-2 receptor) | | | |
|  | ITGA3 | -4.33893 | integrin, alpha 3 (antigen CD49C, alpha 3 subunit of VLA-3 receptor) | | | |
|  | ITGA4 | 22.6432 | integrin, alpha 4 (antigen CD49D, alpha 4 subunit of VLA-4 receptor) | | | |
|  | ITGA6 | 3.60386 | integrin, alpha 6 | | |  |
|  | ITGA8 | 15.1314 | integrin, alpha 8 | | |  |
|  | ITGB4 | -4.59781 | integrin, beta 4 | | |  |
|  | ITGB5 | -3.89526 | integrin, beta 5 | | |  |
|  | ITGB6 | -27.4734 | integrin, beta 6 | | |  |
|  | LAMA1 | 3.89938 | laminin, alpha 1 | | |  |
|  | LAMA3 | 2.06781 | laminin, alpha 3 | | |  |
|  | LAMA4 | 26.1891 | laminin, alpha 4 | | |  |
|  | LAMB1 | 5.53191 | laminin, beta 1 | | |  |
|  | LAMB3 | -2.20262 | laminin, beta 3 | | |  |
|  | LAMC2 | -3.54862 | laminin, gamma 2 | | |  |
|  | SDC1 | -3.29802 | syndecan 1 |  | |  |
|  | SDC4 | -4.72484 | syndecan 4 |  | |  |
|  | SV2A | 3.7792 | synaptic vesicle glycoprotein 2A | | | |
|  | THBS1 | -45.0525 | thrombospondin 1 | | |  |
|  | TNC | 2.28918 | tenascin C |  | |  |
|  |  |  |  |  | |  |
| Tight junction | |  |  |  | |  |
|  | ACTN1 | -2.8211 | actinin, alpha 1 | | |  |
|  | ACTN2 | 2.5575 | actinin, alpha 2 | | |  |
|  | AKT1 | -2.75982 | v-akt murine thymoma viral oncogene homolog 1 (EC:2.7.11.1) | | | |
|  | AKT3 | 86.282 | v-akt murine thymoma viral oncogene homolog 3 (protein kinase B, gamma) (EC:2.7.11.1) | | | |
|  | CASK | -2.27972 | calcium/calmodulin-dependent serine protein kinase (MAGUK family) | | | |
|  | CLDN1 | -3.5734 | claudin 1 |  | |  |
|  | CLDN3 | -6.27181 | claudin 3 |  | |  |
|  | CLDN4 | -9.77984 | claudin 4 |  | |  |
|  | CLDN7 | -21.8632 | claudin 7 |  | |  |
|  | EPB41 | 3.24939 | erythrocyte membrane protein band 4.1 (elliptocytosis 1, RH-linked) | | | |
|  | EPB41L1 | -2.24098 | erythrocyte membrane protein band 4.1-like 1 | | | |
|  | EPB41L2 | 7.54439 | erythrocyte membrane protein band 4.1-like 2 | | | |
|  | EPB41L3 | 21.4853 | erythrocyte membrane protein band 4.1-like 3 | | | |
|  | EXOC4 | 2.01865 | exocyst complex component 4 | | | |
|  | F11R | -2.293 | F11 receptor |  | |  |
|  | GNAI1 | 35.2329 | guanine nucleotide binding protein (G protein), alpha inhibiting | | | |
|  | INADL | -2.31702 | InaD-like (Drosophila) | | |  |
|  | JAM2 | 4.8544 | junctional adhesion molecule 2 | | | |
|  | JAM3 | 15.2062 | junctional adhesion molecule 3 | | | |
|  | LLGL2 | -5.07004 | lethal giant larvae homolog 2 (Drosophila) | | | |
|  | MLLT4 | 3.76221 | myeloid/lymphoid or mixed-lineage leukemia (trithorax homolog, Drosophila); translocated to, 4 | | | |
|  | MPDZ | 2.37926 | multiple PDZ domain protein | | | |
|  | MRAS | 2.8653 | muscle RAS oncogene homolog | | | |
|  | MYH10 | 3.38841 | myosin, heavy chain 10, non-muscle | | | |
|  | MYH14 | -2.38333 | myosin, heavy chain 14 | | |  |
|  | NRAS | -2.4873 | neuroblastoma RAS viral (v-ras) oncogene homolog | | | |
|  | PARD6B | -10.1306 | par-6 partitioning defective 6 homolog beta (C. elegans) | | | |
|  | PPP2R2C | -6.94589 | protein phosphatase 2 (formerly 2A), regulatory subunit B, gamma | | | |
|  | PRKCA | 2.00421 | protein kinase C, alpha (EC:2.7.11.13) | | | |
|  | PRKCB | 2.17984 | protein kinase C, beta (EC:2.7.11.13) | | | |
|  | PRKCD | -2.07442 | protein kinase C, delta (EC:2.7.11.13) | | | |
|  | PRKCE | -2.07196 | protein kinase C, epsilon (EC:2.7.11.13) | | | |
|  | PRKCQ | 3.56236 | protein kinase C, theta (EC:2.7.11.13) | | | |
|  | RAB3B | 2.47895 | RAB3B, member RAS oncogene family | | | |
|  | TJP3 | -2.50594 | tight junction protein 3 (zona occludens 3) | | | |
|  |  |  |  |  | |  |
|  |  |  |  |  | |  |
| Regulation of actin cytoskeleton | | |  |  | |  |
|  | ACTN1 | -2.8211 | actinin, alpha 1 | | |  |
|  | ACTN2 | 2.5575 | actinin, alpha 2 | | |  |
|  | ARHGEF6 | 8.91873 | Rac/Cdc42 guanine nucleotide exchange factor (GEF) 6 | | | |
|  | ARPC1B | -4.96183 | actin related protein 2/3 complex, subunit 1B, 41kDa | | | |
|  | BAIAP2 | -2.13381 | BAI1-associated protein 2 | | | |
|  | BDKRB2 | -2.81651 | bradykinin receptor B2 | | |  |
|  | CFL2 | 3.63673 | cofilin 2 (muscle) | | |  |
|  | CYFIP2 | 4.7318 | cytoplasmic FMR1 interacting protein 2 | | | |
|  | DIAPH2 | 2.59683 | diaphanous homolog 2 (Drosophila) | | | |
|  | DIAPH3 | 2.78478 | diaphanous homolog 3 (Drosophila) | | | |
|  | ENAH | 2.21826 | enabled homolog (Drosophila) | | | |
|  | F2R | 8.3542 | coagulation factor II (thrombin) receptor | | | |
|  | FGF13 | 3.43852 | fibroblast growth factor 13 | | | |
|  | FGF2 | 3.23962 | fibroblast growth factor 2 (basic) | | | |
|  | FGF5 | 2.26717 | fibroblast growth factor 5 | | | |
|  | FGF9 | 7.08775 | fibroblast growth factor 9 (glia-activating factor) | | | |
|  | FGFR1 | 2.06284 | fibroblast growth factor receptor 1 (EC:2.7.10.1) | | | |
|  | FGFR2 | 2.69989 | fibroblast growth factor receptor 2 (EC:2.7.10.1) | | | |
|  | FN1 | -2.61255 | fibronectin 1 |  | |  |
|  | GNG12 | 2.24456 | guanine nucleotide binding protein (G protein), gamma 12 | | | |
|  | GSN | -2.36136 | gelsolin (amyloidosis, Finnish type) | | | |
|  | IQGAP2 | 48.5857 | IQ motif containing GTPase activating protein 2 | | | |
|  | ITGA2 | -4.45339 | integrin, alpha 2 (CD49B, alpha 2 subunit of VLA-2 receptor) | | | |
|  | ITGA3 | -4.33893 | integrin, alpha 3 (antigen CD49C, alpha 3 subunit of VLA-3 receptor) | | | |
|  | ITGA4 | 22.6432 | integrin, alpha 4 (antigen CD49D, alpha 4 subunit of VLA-4 receptor) | | | |
|  | ITGA6 | 3.60386 | integrin, alpha 6 | | |  |
|  | ITGA8 | 15.1314 | integrin, alpha 8 | | |  |
|  | ITGB4 | -4.59781 | integrin, beta 4 | | |  |
|  | ITGB5 | -3.89526 | integrin, beta 5 | | |  |
|  | ITGB6 | -27.4734 | integrin, beta 6 | | |  |
|  | MRAS | 2.8653 | muscle RAS oncogene homolog | | | |
|  | MSN | 21.1665 | moesin |  | |  |
|  | MYH10 | 3.38841 | myosin, heavy chain 10, non-muscle | | | |
|  | MYH14 | -2.38333 | myosin, heavy chain 14 | | |  |
|  | MYLK | -3.96761 | myosin light chain kinase (EC:2.7.11.18) | | | |
|  | NRAS | -2.4873 | neuroblastoma RAS viral (v-ras) oncogene homolog | | | |
|  | PAK1 | 5.15964 | p21 protein (Cdc42/Rac)-activated kinase 1 (EC:2.7.11.1) | | | |
|  | PDGFA | -2.22123 | platelet-derived growth factor alpha polypeptide | | | |
|  | PDGFC | 11.677 | platelet derived growth factor C | | | |
|  | PDGFD | 3.45827 | platelet derived growth factor D | | | |
|  | PDGFRA | 2.27842 | platelet-derived growth factor receptor, alpha polypeptide | | | |
|  | PIK3CB | -2.34109 | phosphoinositide-3-kinase, catalytic, beta polypeptide | | | |
|  | PIK3R1 | -2.03668 | phosphoinositide-3-kinase, regulatory subunit 1 (alpha) | | | |
|  | PIP4K2C | -2.8568 | phosphatidylinositol-5-phosphate 4-kinase, type II, gamma | | | |
|  | PXN | -2.69475 | paxillin |  | |  |
|  | RAC3 | 2.73518 | ras-related C3 botulinum toxin substrate 3 (rho family, small GTP | | | |
|  | SSH3 | -2.09543 | slingshot homolog 3 (Drosophila) (EC:3.1.3.16 3.1.3.48) | | | |
|  | TIAM2 | 5.59659 | T-cell lymphoma invasion and metastasis 2 | | | |
|  | TMSB4X | -7.3656 | thymosin beta 4, X-linked | | | |
|  | TMSL3 | -6.22906 | thymosin-like 3 | | |  |
|  | VAV3 | -3.18735 | vav 3 guanine nucleotide exchange factor | | | |
|  | WASF1 | 4.43784 | WAS protein family, member 1 | | | |
|  |  |  |  |  | |  |
| ErbB signaling pathway | | |  |  | |  |
|  | AKT1 | -2.75982 | v-akt murine thymoma viral oncogene homolog 1 (EC:2.7.11.1) | | | |
|  | AKT3 | 86.282 | v-akt murine thymoma viral oncogene homolog 3 (protein kinase B, gamma) (EC:2.7.11.1) | | | |
|  | AREG | -134.276 | amphiregulin |  | |  |
|  | BTC | -3.65095 | betacellulin |  | |  |
|  | CAMK2D | 3.4734 | calcium/calmodulin-dependent protein kinase II delta (EC:2.7.11.17) | | | |
|  | ERBB3 | -5.16774 | v-erb-b2 erythroblastic leukemia viral oncogene homolog 3 (avian) | | | |
|  | ERBB4 | 3.03933 | v-erb-a erythroblastic leukemia viral oncogene homolog 4 (avian) | | | |
|  | MAPK8 | 2.03382 | mitogen-activated protein kinase 8 (EC:2.7.11.24) | | | |
|  | NRAS | -2.4873 | neuroblastoma RAS viral (v-ras) oncogene homolog | | | |
|  | NRG4 | 2.80689 | neuregulin 4 |  | |  |
|  | PAK1 | 5.15964 | p21 protein (Cdc42/Rac)-activated kinase 1 (EC:2.7.11.1) | | | |
|  | PIK3CB | -2.34109 | phosphoinositide-3-kinase, catalytic, beta polypeptide | | | |
|  | PIK3R1 | -2.03668 | phosphoinositide-3-kinase, regulatory subunit 1 (alpha) | | | |
|  | PLCG1 | -2.56212 | phospholipase C, gamma 1 (EC:3.1.4.11) | | | |
|  | PRKCA | 2.00421 | protein kinase C, alpha (EC:2.7.11.13) | | | |
|  | PRKCB | 2.17984 | protein kinase C, beta (EC:2.7.11.13) | | | |
|  | RPS6KB1 | -3.74595 | ribosomal protein S6 kinase, 70kDa, polypeptide 1 (EC:2.7.11.1) | | | |
|  | SHC4 | -3.80376 | SHC (Src homology 2 domain containing) family, member 4 | | | |
|  | TGFA | -4.90783 | transforming growth factor, alpha | | | |
|  |  |  |  |  | |  |
| p53 signaling pathway | | |  |  | |  |
|  | APAF1 | 3.285 | apoptotic peptidase activating factor 1 | | | |
|  | ATM | 3.03337 | ataxia telangiectasia mutated (EC:2.7.11.1) | | | |
|  | CASP3 | 3.41838 | caspase 3, apoptosis-related cysteine peptidase (EC:3.4.22.56) | | | |
|  | CASP8 | -2.04954 | caspase 8, apoptosis-related cysteine peptidase (EC:3.4.22.61) | | | |
|  | CCND1 | -7.59907 | cyclin D1 |  | |  |
|  | CCND2 | 30.8703 | cyclin D2 |  | |  |
|  | CCNG1 | 3.0795 | cyclin G1 |  | |  |
|  | CDK6 | 6.74438 | cyclin-dependent kinase 6 (EC:2.7.11.22) | | | |
|  | CDKN2A | 10.6198 | cyclin-dependent kinase inhibitor 2A (melanoma, p16, inhibits CDK4) | | | |
|  | FAS | 2.23839 | Fas (TNF receptor superfamily, member 6) | | | |
|  | PMAIP1 | 6.65897 | phorbol-12-myristate-13-acetate-induced protein 1 | | | |
|  | PPM1D | -3.20037 | protein phosphatase 1D magnesium-dependent, delta isoform | | | |
|  | RPRM | 2.69107 | reprimo, TP53 dependent G2 arrest mediator candidate | | | |
|  | SESN1 | 4.07654 | sestrin 1 |  | |  |
|  | SESN2 | 2.11551 | sestrin 2 |  | |  |
|  | SESN3 | 7.2855 | sestrin 3 |  | |  |
|  | SFN | -10.8867 | stratifin |  | |  |
|  | THBS1 | -45.0525 | thrombospondin 1 | | |  |
|  | TNFRSF10B | 3.3647 | tumor necrosis factor receptor superfamily, member 10b | | | |
|  | TP53I3 | -3.21944 | tumor protein p53 inducible protein 3 | | | |
|  | ZMAT3 | 2.53994 | zinc finger, matrin type 3 | | | |
|  |  |  |  |  | |  |
| Apoptosis | |  |  |  | |  |
|  | AIFM1 | 2.39869 | apoptosis-inducing factor, mitochondrion-associated, 1 | | | |
|  | AKT1 | -2.75982 | v-akt murine thymoma viral oncogene homolog 1 (EC:2.7.11.1) | | | |
|  | AKT3 | 86.282 | v-akt murine thymoma viral oncogene homolog 3 (protein kinase B, gamma) (EC:2.7.11.1) | | | |
|  | APAF1 | 3.285 | apoptotic peptidase activating factor 1 | | | |
|  | ATM | 3.03337 | ataxia telangiectasia mutated (EC:2.7.11.1) | | | |
|  | BCL2 | -3.16498 | B-cell CLL/lymphoma 2 | | | |
|  | BCL2L1 | -3.13264 | BCL2-like 1 |  | |  |
|  | CAPN1 | -2.0789 | calpain 1, (mu/I) large subunit (EC:3.4.22.52) | | | |
|  | CAPN2 | 2.17305 | calpain 2, (m/II) large subunit (EC:3.4.22.53) | | | |
|  | CASP10 | 2.02778 | caspase 10, apoptosis-related cysteine peptidase (EC:3.4.22.63) | | | |
|  | CASP3 | 3.41838 | caspase 3, apoptosis-related cysteine peptidase (EC:3.4.22.56) | | | |
|  | CASP8 | -2.04954 | caspase 8, apoptosis-related cysteine peptidase (EC:3.4.22.61) | | | |
|  | FAS | 2.23839 | Fas (TNF receptor superfamily, member 6) | | | |
|  | MAP3K14 | -2.61877 | mitogen-activated protein kinase kinase kinase 14 (EC:2.7.11.25) | | | |
|  | MYD88 | -3.27423 | myeloid differentiation primary response gene (88) | | | |
|  | NFKBIA | -3.54182 | nuclear factor of kappa light polypeptide gene enhancer in B-cells | | | |
|  | PIK3CB | -2.34109 | phosphoinositide-3-kinase, catalytic, beta polypeptide | | | |
|  | PIK3R1 | -2.03668 | phosphoinositide-3-kinase, regulatory subunit 1 (alpha) | | | |
|  | PRKACB | 2.65663 | protein kinase, cAMP-dependent, catalytic, beta (EC:2.7.11.11) | | | |
|  | PRKAR1B | -2.36979 | protein kinase, cAMP-dependent, regulatory, type I, beta | | | |
|  | TNFRSF10B | 3.3647 | tumor necrosis factor receptor superfamily, member 10b | | | |
|  | TNFRSF10D | 17.2915 | tumor necrosis factor receptor superfamily, member 10d, decoy with | | | |
|  | TNFRSF1A | -2.06155 | tumor necrosis factor receptor superfamily, member 1A | | | |
|  |  |  |  |  | |  |
| Phosphatidylinositol signaling system | | |  |  | |  |
|  | CALM1 | -2.05357 | calmodulin 1 (phosphorylase kinase, delta) (EC:2.7.11.19) | | | |
|  | CALM2 | -2.11346 | calmodulin 2 (phosphorylase kinase, delta) | | | |
|  | IMPA1 | 3.04099 | inositol(myo)-1(or 4)-monophosphatase 1 (EC:3.1.3.25) | | | |
|  | INPP1 | 3.77104 | inositol polyphosphate-1-phosphatase (EC:3.1.3.57) | | | |
|  | INPP4B | -14.8536 | inositol polyphosphate-4-phosphatase, type II, 105kDa (EC:3.1.3.66) | | | |
|  | INPP5B | 2.69118 | inositol polyphosphate-5-phosphatase, 75kDa (EC:3.1.3.36) | | | |
|  | INPP5D | 2.80564 | inositol polyphosphate-5-phosphatase, 145kDa | | | |
|  | ITPK1 | -3.44772 | inositol 1,3,4-triphosphate 5/6 kinase (EC:2.7.1.134 2.7.1.159) | | | |
|  | ITPR1 | 2.13683 | inositol 1,4,5-triphosphate receptor, type 1 | | | |
|  | OCRL | 2.68981 | oculocerebrorenal syndrome of Lowe (EC:3.1.3.36) | | | |
|  | PIK3C2B | -2.57937 | phosphoinositide-3-kinase, class 2, beta polypeptide (EC:2.7.1.154) | | | |
|  | PIK3C3 | 3.33325 | phosphoinositide-3-kinase, class 3 (EC:2.7.1.137) | | | |
|  | PIK3CB | -2.34109 | phosphoinositide-3-kinase, catalytic, beta polypeptide | | | |
|  | PIK3R1 | -2.03668 | phosphoinositide-3-kinase, regulatory subunit 1 (alpha) | | | |
|  | PIP4K2C | -2.8568 | phosphatidylinositol-5-phosphate 4-kinase, type II, gamma | | | |
|  | PLCB4 | -2.06443 | phospholipase C, beta 4 (EC:3.1.4.11) | | | |
|  | PLCE1 | 14.4462 | phospholipase C, epsilon 1 (EC:3.1.4.11) | | | |
|  | PLCG1 | -2.56212 | phospholipase C, gamma 1 (EC:3.1.4.11) | | | |
|  | PRKCA | 2.00421 | protein kinase C, alpha (EC:2.7.11.13) | | | |
|  | PRKCB | 2.17984 | protein kinase C, beta (EC:2.7.11.13) | | | |
|  |  |  |  |  | |  |
| Gap junction | |  |  |  | |  |
|  | ADCY5 | -2.91719 | adenylate cyclase 5 (EC:4.6.1.1) | | | |
|  | GJA1 | 12.383 | gap junction protein, alpha 1, 43kDa | | | |
|  | GNAI1 | 35.2329 | guanine nucleotide binding protein (G protein), alpha inhibiting | | | |
|  | GUCY1A2 | 3.9613 | guanylate cyclase 1, soluble, alpha 2 (EC:4.6.1.2) | | | |
|  | GUCY1A3 | 8.18999 | guanylate cyclase 1, soluble, alpha 3 (EC:4.6.1.2) | | | |
|  | GUCY1B3 | 14.2834 | guanylate cyclase 1, soluble, beta 3 (EC:4.6.1.2) | | | |
|  | ITPR1 | 2.13683 | inositol 1,4,5-triphosphate receptor, type 1 | | | |
|  | LPAR1 | 13.1777 | lysophosphatidic acid receptor 1 | | | |
|  | MAP2K5 | 2.55893 | mitogen-activated protein kinase kinase 5 (EC:2.7.12.2) | | | |
|  | NRAS | -2.4873 | neuroblastoma RAS viral (v-ras) oncogene homolog | | | |
|  | PDGFA | -2.22123 | platelet-derived growth factor alpha polypeptide | | | |
|  | PDGFC | 11.677 | platelet derived growth factor C | | | |
|  | PDGFD | 3.45827 | platelet derived growth factor D | | | |
|  | PDGFRA | 2.27842 | platelet-derived growth factor receptor, alpha polypeptide | | | |
|  | PLCB4 | -2.06443 | phospholipase C, beta 4 (EC:3.1.4.11) | | | |
|  | PRKACB | 2.65663 | protein kinase, cAMP-dependent, catalytic, beta (EC:2.7.11.11) | | | |
|  | PRKCA | 2.00421 | protein kinase C, alpha (EC:2.7.11.13) | | | |
|  | PRKCB | 2.17984 | protein kinase C, beta (EC:2.7.11.13) | | | |
|  | PRKG1 | 14.9216 | protein kinase, cGMP-dependent, type I (EC:2.7.11.12) | | | |
|  | TUBA1C | -2.85695 | tubulin, alpha 1c | | |  |
|  | TUBB2B | 11.8669 | tubulin, beta 2B | | |  |
|  |  |  |  |  | |  |
| TGF-beta signaling pathway | | |  |  | |  |
|  | ACVR2B | 2.74239 | activin A receptor, type IIB (EC:2.7.11.30) | | | |
|  | BMP6 | 2.80212 | bone morphogenetic protein 6 | | | |
|  | BMP7 | -3.20791 | bone morphogenetic protein 7 | | | |
|  | ID2 | 2.10873 | inhibitor of DNA binding 2, dominant negative helix-loop-helix | | | |
|  | ID4 | 5.84311 | inhibitor of DNA binding 4, dominant negative helix-loop-helix | | | |
|  | INHBA | -2.26279 | inhibin, beta A | | |  |
|  | RPS6KB1 | -3.74595 | ribosomal protein S6 kinase, 70kDa, polypeptide 1 (EC:2.7.11.1) | | | |
|  | SMAD2 | 2.13231 | SMAD family member 2 | | | |
|  | SMAD3 | -2.58977 | SMAD family member 3 | | | |
|  | SMAD4 | 2.76934 | SMAD family member 4 | | | |
|  | SMAD9 | 3.67452 | SMAD family member 9 | | | |
|  | SMURF1 | -2.50515 | SMAD specific E3 ubiquitin protein ligase 1 | | | |
|  | TFDP1 | 2.47964 | transcription factor Dp-1 | | | |
|  | TGFB1 | -2.2187 | transforming growth factor, beta 1 | | | |
|  | TGFB2 | -4.55364 | transforming growth factor, beta 2 | | | |
|  | THBS1 | -45.0525 | thrombospondin 1 | | |  |
|  | ZFYVE9 | 2.72623 | zinc finger, FYVE domain containing 9 | | | |
|  |  |  |  |  | |  |
|  |  |  |  |  | |  |
| mTOR signaling pathway | | |  |  | |  |
|  | AKT1 | -2.75982 | v-akt murine thymoma viral oncogene homolog 1 (EC:2.7.11.1) | | | |
|  | AKT3 | 86.282 | v-akt murine thymoma viral oncogene homolog 3 (protein kinase B, gamma) (EC:2.7.11.1) | | | |
|  | PDPK1 | -2.00895 | 3-phosphoinositide dependent protein kinase-1 (EC:2.7.11.1) | | | |
|  | PGF | 2.69592 | placental growth factor | | |  |
|  | PIK3CB | -2.34109 | phosphoinositide-3-kinase, catalytic, beta polypeptide | | | |
|  | PIK3R1 | -2.03668 | phosphoinositide-3-kinase, regulatory subunit 1 (alpha) | | | |
|  | PRKAA2 | 7.70806 | protein kinase, AMP-activated, alpha 2 catalytic subunit | | | |
|  | RICTOR | 2.16568 | rapamycin-insensitive companion of mTOR | | | |
|  | RPS6KA6 | 4.21396 | ribosomal protein S6 kinase, 90kDa, polypeptide 6 (EC:2.7.11.1) | | | |
|  | RPS6KB1 | -3.74595 | ribosomal protein S6 kinase, 70kDa, polypeptide 1 (EC:2.7.11.1) | | | |
|  |  |  |  |  | |  |
| VEGF signaling pathway | | |  |  | |  |
|  | AKT1 | -2.75982 | v-akt murine thymoma viral oncogene homolog 1 (EC:2.7.11.1) | | | |
|  | AKT3 | 86.282 | v-akt murine thymoma viral oncogene homolog 3 (protein kinase B, gamma) (EC:2.7.11.1) | | | |
|  | HSPB1 | -6.39327 | heat shock 27kDa protein 1 | | | |
|  | MAPKAPK2 | -3.37289 | mitogen-activated protein kinase-activated protein kinase 2 | | | |
|  | NFATC2 | -12.9791 | nuclear factor of activated T-cells, cytoplasmic, calcineurin-dependent 2 | | | |
|  | NRAS | -2.4873 | neuroblastoma RAS viral (v-ras) oncogene homolog | | | |
|  | PIK3CB | -2.34109 | phosphoinositide-3-kinase, catalytic, beta polypeptide | | | |
|  | PIK3R1 | -2.03668 | phosphoinositide-3-kinase, regulatory subunit 1 (alpha) | | | |
|  | PLA2G10 | -2.69651 | phospholipase A2, group X (EC:3.1.1.4) | | | |
|  | PLA2G4A | 14.5133 | phospholipase A2, group IVA (cytosolic, calcium-dependent) | | | |
|  | PLCG1 | -2.56212 | phospholipase C, gamma 1 (EC:3.1.4.11) | | | |
|  | PRKCA | 2.00421 | protein kinase C, alpha (EC:2.7.11.13) | | | |
|  | PRKCB | 2.17984 | protein kinase C, beta (EC:2.7.11.13) | | | |
|  | PXN | -2.69475 | paxillin |  | |  |
|  | RAC3 | 2.73518 | ras-related C3 botulinum toxin substrate 3 (rho family, small GTP | | | |
|  | SPHK1 | -2.66028 | sphingosine kinase 1 (EC:2.7.1.91) | | | |
|  |  |  |  |  | |  |
| Cell cycle |  |  |  |  | |  |
|  | ATM | 3.03337 | ataxia telangiectasia mutated (EC:2.7.11.1) | | | |
|  | CCND1 | -7.59907 | cyclin D1 |  | |  |
|  | CCND2 | 30.8703 | cyclin D2 |  | |  |
|  | CDC14B | 2.65057 | CDC14 cell division cycle 14 homolog B (S. cerevisiae) (EC:3.1.3.16 | | | |
|  | CDC16 | 2.58102 | cell division cycle 16 homolog (S. cerevisiae) | | | |
|  | CDC23 | 2.12771 | cell division cycle 23 homolog (S. cerevisiae) | | | |
|  | CDC25A | 2.2013 | cell division cycle 25 homolog A (S. pombe) (EC:3.1.3.48) | | | |
|  | CDC25B | -2.44609 | cell division cycle 25 homolog B (S. pombe) (EC:3.1.3.48) | | | |
|  | CDC25C | 2.37618 | cell division cycle 25 homolog C (S. pombe) (EC:3.1.3.48) | | | |
|  | CDC7 | 3.30004 | cell division cycle 7 homolog (S. cerevisiae) (EC:2.7.11.1) | | | |
|  | CDK6 | 6.74438 | cyclin-dependent kinase 6 (EC:2.7.11.22) | | | |
|  | CDKN2A | 10.6198 | cyclin-dependent kinase inhibitor 2A (melanoma, p16, inhibits CDK4) | | | |
|  | CDKN2C | 2.89832 | cyclin-dependent kinase inhibitor 2C (p18, inhibits CDK4) | | | |
|  | MCM3 | 2.08625 | minichromosome maintenance complex component 3 | | | |
|  | PKMYT1 | -2.09018 | protein kinase, membrane associated tyrosine/threonine 1 | | | |
|  | SFN | -10.8867 | stratifin |  | |  |
|  | SKP2 | 5.15539 | S-phase kinase-associated protein 2 (p45) | | | |
|  | SMAD2 | 2.13231 | SMAD family member 2 | | | |
|  | SMAD3 | -2.58977 | SMAD family member 3 | | | |
|  | SMAD4 | 2.76934 | SMAD family member 4 | | | |
|  | SMC1A | 2.47693 | structural maintenance of chromosomes 1A | | | |
|  | TFDP1 | 2.47964 | transcription factor Dp-1 | | | |
|  | TGFB1 | -2.2187 | transforming growth factor, beta 1 | | | |
|  | TGFB2 | -4.55364 | transforming growth factor, beta 2 | | | |
|  | WEE1 | 2.01502 | WEE1 homolog (S. pombe) (EC:2.7.10.2) | | | |
|  |  |  |  |  | |  |
| ABC transporters | |  |  |  | |  |
|  | ABCA12 | -30.567 | ATP-binding cassette, sub-family A (ABC1), member 12 | | | |
|  | ABCB1 | 29.8734 | ATP-binding cassette, sub-family B (MDR/TAP), member 1 (EC:3.6.3.44) | | | |
|  | ABCB4 | 3.47448 | ATP-binding cassette, sub-family B (MDR/TAP), member 4 (EC:3.6.3.44) | | | |
|  | ABCB6 | -2.84869 | ATP-binding cassette, sub-family B (MDR/TAP), member 6 | | | |
|  | ABCB7 | 2.38133 | ATP-binding cassette, sub-family B (MDR/TAP), member 7 | | | |
|  | ABCC2 | -5.47011 | ATP-binding cassette, sub-family C (CFTR/MRP), member 2 | | | |
|  | ABCC3 | -7.05971 | ATP-binding cassette, sub-family C (CFTR/MRP), member 3 | | | |
|  | ABCC4 | 2.61441 | ATP-binding cassette, sub-family C (CFTR/MRP), member 4 | | | |
|  | ABCD2 | 3.99446 | ATP-binding cassette, sub-family D (ALD), member 2 | | | |
|  | ABCD3 | 2.38568 | ATP-binding cassette, sub-family D (ALD), member 3 | | | |
|  | ABCG2 | -4.66654 | ATP-binding cassette, sub-family G (WHITE), member 2 | | | |
