# Supplementary material for: MEK5/ERK5 Signaling Suppresses Estrogen Receptor Expression and Promotes Hormone-Independent Tumorigenesis
Source: PLoS One. 2013 Aug 9;8(8):e69291. doi: 10.1371/journal.pone.0069291 (PMC3739787; doi:10.1371/journal.pone.0069291)
Supplement: Table S2 — MEK5 induced EMT gene expression changes. (DOCX) [file pone.0069291.s006.docx]

**Supplemental Table 2. EMT Gene Expression Changes Associated with MEK5 Expression (p<0.05 for all listed genes)**

| *Gene Symbol* | *Fold Change (MEK5 vs. VEC)* | *p-value* |
| --- | --- | --- |
| SUSD5 | 2.33 | 3.34E-05 |
| SAMD9 | -1.65 | 2.04E-03 |
| CCDC88A | 28.43 | 3.27E-11 |
| CCDC99 | 1.72 | 4.67E-05 |
| FLJ32810 | 8.27 | 1.55E-07 |
| IFI44 | -1.56 | 1.74E-04 |
| DDX60 | -3.29 | 1.62E-05 |
| IGF2BP3 | 21.19 | 1.13E-05 |
| PAG1 | 4.53 | 6.69E-06 |
| CCDC82 | 14.16 | 3.23E-08 |
| ANXA1 | 2.58 | 1.15E-04 |
| IMPA1 | 3.04 | 2.75E-07 |
| LYST | 2.96 | 6.60E-07 |
| KIAA1524 | -1.05 | 3.39E-01 |
| ANP32E | 1.58 | 3.30E-04 |
| BIRC3 | -1.53 | 1.40E-03 |
| DNAJB4 | 3.03 | 6.70E-08 |
| LYN | 8.71 | 9.53E-09 |
| CD83 | 1.12 | 2.29E-02 |
| OSTM1 | 1.26 | 3.31E-02 |
| PMAIP1 | 6.66 | 5.27E-08 |
| CHN1 | 1.56 | 7.11E-04 |
| MYBL1 | -2.34 | 2.62E-05 |
| RAD18 | -1.04 | 4.51E-01 |
| AGPAT5 | 1.17 | 4.23E-03 |
| ARSJ | 3.65 | 5.20E-06 |
| C6orf173 | 3.57 | 4.74E-06 |
| GULP1 | -1.15 | 1.40E-01 |
| DCBLD2 | 1.19 | 7.35E-04 |
| HMGA2 | 3.88 | 1.57E-05 |
| MAK16 | -1.09 | 3.27E-01 |
| DPH3 | 1.65 | 1.94E-04 |
| SMC5 | 1.28 | 3.27E-03 |
| PBK | 1.45 | 8.69E-04 |
| CTNNAL1 | 1.53 | 5.34E-04 |
| UBLCP1 | 2.17 | 7.65E-04 |
| TTK | 2.28 | 9.22E-05 |
| LIFR | 5.54 | 6.97E-08 |
| SOAT1 | 1.77 | 2.98E-04 |
| PPARG | 1.14 | 3.86E-02 |
| MGAT5B | -1.14 | 2.03E-03 |
| ABCC4 | 2.61 | 3.18E-07 |
| NOL8 | -1.1 | 1.41E-01 |
| ACSL4 | 21.43 | 1.07E-10 |
| HJURP | -2.64 | 8.39E-08 |
| KIAA0020 | -1.53 | 4.51E-04 |
| POT1 | 3.13 | 3.51E-06 |
| AP1S2 | 3.85 | 1.76E-06 |
| CLSPN | 1.92 | 1.99E-06 |
| ANLN | 1.16 | 5.76E-02 |
| FAM92A1 | 1.3 | 1.96E-02 |
| CAMK4 | 14.47 | 7.48E-07 |
| E2F7 | 1.89 | 3.16E-06 |
| GBP1 | -2.92 | 1.26E-05 |
| GNAI1 | 35.23 | 6.41E-09 |
| FABP5 | 1.75 | 1.81E-03 |
| CKS2 | 1.35 | 2.38E-03 |
| PTTG3 | 1.14 | 1.38E-01 |
| IFIT2 | -4.33 | 5.83E-05 |
| ECHDC1 | 1.71 | 2.91E-04 |
| FAM40B | 2.53 | 1.97E-05 |
| ZMYM1 | 1.27 | 1.68E-03 |
| RB1CC1 | 1.66 | 6.21E-04 |
| COMMD8 | 1.56 | 7.94E-04 |
| SACS | 5.05 | 1.10E-07 |
| GALNT1 | 1.09 | 3.05E-01 |
| PTTG1 | 1.69 | 2.23E-05 |
| C2orf44 | 1.39 | 3.34E-03 |
| RP2 | 2.14 | 5.17E-05 |
| LACTB | -1.84 | 9.25E-05 |
| SCML1 | 2.63 | 2.89E-06 |
| WDR47 | -1.36 | 1.59E-02 |
| IKIP | 2.58 | 4.42E-06 |
| MAP7D3 | 4.66 | 9.40E-07 |
| HDAC9 | 2.02 | 1.50E-05 |
| RND3 | -1.84 | 6.95E-05 |
| PPM2C | -1.03 | 3.18E-02 |
| CLDND1 | 1.74 | 2.57E-06 |
| DDX60L | -4.33 | 2.89E-08 |
| DNER | -1.24 | 9.94E-03 |
| ZNF788 | -1.05 | 3.39E-01 |
| MCAM | 2.04 | 1.06E-04 |
| PNMA2 | 9.99 | 4.27E-07 |
| USP33 | 2.61 | 6.79E-09 |
| C6orf150 | -20.34 | 1.21E-08 |
| WDR19 | 4.43 | 1.12E-06 |
| TTC27 | -1.71 | 2.60E-04 |
| POLK | 2.5 | 4.25E-06 |
| ARL4A | -1.03 | 4.19E-01 |
| ERBB3 | -5.17 | 3.03E-07 |
| C1orf172 | -2.2 | 2.35E-06 |
| MAL2 | -90.7 | 2.72E-11 |
| TSPAN13 | -2.76 | 1.00E-07 |
| GRHL2 | -48.45 | 6.80E-10 |
| SPINT1 | -5.96 | 4.26E-08 |
| ST14 | -13.89 | 4.15E-08 |
| ATCAY | -1.01 | 5.23E-01 |
| S100A14 | -12.28 | 4.43E-07 |
| PROM2 | -2.07 | 5.55E-05 |
| PRSS22 | -2.02 | 3.31E-05 |
| CD24 | -17.33 | 8.07E-08 |
| ANXA9 | -17.43 | 3.98E-08 |
| CLDN7 | -21.86 | 1.08E-08 |
| ABCA12 | -30.57 | 7.91E-12 |
| MREG | -5.98 | 4.37E-07 |
| TSPAN15 | -3.09 | 1.60E-06 |
| MPZL3 | -3.33 | 3.00E-07 |
| SPINT2 | -21.78 | 7.72E-09 |
| EFNA1 | -3.62 | 1.38E-06 |
| TACSTD2 | -17.4 | 6.62E-08 |
| MYH14 | -2.38 | 1.52E-04 |
| EVPL | -1.6 | 8.07E-05 |
| TSPAN1 | -5.74 | 5.00E-07 |
| PRKCH | -1.41 | 6.37E-04 |
| PPM1L | 1.5 | 3.96E-05 |
| SLC9A3R1 | -5.57 | 2.96E-07 |
| GRHL1 | -2.72 | 1.54E-07 |
| MARVELD2 | -2.52 | 2.61E-06 |
| CLDN3 | -6.27 | 1.28E-06 |
| YBX2 | -1.54 | 7.72E-05 |
| SYNE2 | -1.11 | 2.16E-02 |
| TBC1D30 | -5.78 | 5.83E-07 |
| ELF3 | -7.52 | 6.22E-07 |
| AGR2 | -65.6 | 7.90E-10 |
| OCLN | -1.43 | 2.13E-04 |
| SELENBP1 | -1.24 | 7.89E-03 |
| ATAD4 | -6.19 | 2.03E-07 |
| RBM47 | -3.88 | 1.73E-07 |
| IGSF3 | -3.21 | 2.93E-06 |
| C17orf28 | -3.64 | 2.96E-06 |
| LLGL2 | -5.07 | 7.66E-07 |
| SPDEF | -13.67 | 2.26E-08 |
| LRRC1 | -1.14 | 5.74E-02 |
| SLC29A2 | -2.53 | 6.06E-07 |
| PKP3 | -1.77 | 5.68E-04 |
| IGSF9 | -1.64 | 5.67E-05 |
| VAMP8 | -6.74 | 5.10E-07 |
| ICA1 | -2.34 | 5.49E-06 |
| CLDN4 | -9.78 | 5.24E-08 |
| MLPH | -15.57 | 1.80E-08 |
| TOM1L1 | 1.25 | 9.83E-03 |
| CEBPA | -1.32 | 6.55E-03 |
| C20orf151 | -1.29 | 1.14E-02 |
| GCA | -1.1 | 3.84E-01 |
| AIM1 | -14.06 | 4.53E-08 |
| KIF21A | -1.3 | 1.19E-03 |
| SH3YL1 | -2.36 | 3.36E-08 |
| MSX2 | -1 | 9.33E-01 |
| CKMT1A | -3.22 | 7.57E-07 |
| MYB | -7.83 | 1.26E-07 |
| L2HGDH | -1.61 | 5.41E-06 |
| GALNT3 | -7.73 | 2.64E-07 |
| C9orf140 | -1.62 | 1.13E-03 |
| RAB25 | -43.32 | 1.28E-08 |
| CDH1 | -46.38 | 1.26E-10 |
| TPD52 | -1.96 | 4.36E-04 |
| PIK3R3 | -1.59 | 2.67E-06 |
| KRT8 | -15.65 | 5.08E-09 |
| LAD1 | -9.67 | 4.67E-07 |
| CELSR1 | -1.28 | 5.58E-02 |
| FXYD3 | -26.84 | 3.59E-08 |
| KIAA1598 | -1.25 | 4.14E-03 |
| LNX2 | -1.95 | 5.34E-06 |
| C10orf58 | -1.26 | 2.27E-03 |
| RP6-213H19.1 | 2 | 1.88E-06 |
| PLEKHF2 | -4.64 | 5.47E-06 |
| MAP3K1 | -1.6 | 1.35E-04 |
| PREX1 | -15.36 | 1.55E-09 |
