# Supplementary material for: MEK5/ERK5 Signaling Suppresses Estrogen Receptor Expression and Promotes Hormone-Independent Tumorigenesis
Source: PLoS One. 2013 Aug 9;8(8):e69291. doi: 10.1371/journal.pone.0069291 (PMC3739787; doi:10.1371/journal.pone.0069291)
Supplement: Table S3 — TNF pathway alterations associated with MEK5 expression. (DOCX) [file pone.0069291.s007.docx]

**Supplemental Table 3. Tumor Necrosis Factor Pathway Gene Expression Changes Associated with MEK5 Expression**

| *Group* | *Gene Symbol* | | *Fold Change* | | *p-value* | |
| --- | --- | --- | --- | --- | --- | --- |
| ***TNF/TNFR*** | | TNFRSF1A | | -2.06 | | 2.57E-05 |
|  |  | TNFRSF19 | | 2.48 | | 6.90E-06 |
|  |  | TNFSF11 | | -1.14 | | 2.52E-02 |
|  |  | TNFSF13B | | 1.18 | | 1.48E-02 |
|  |  | TNFRSF12A | | -8.65 | | 1.90E-07 |
|  |  | TNFRSF17 | | -1.09 | | 3.72E-01 |
|  |  | TNFRSF17 | | -1.31 | | 8.08E-03 |
|  |  | TNFSF12-TNFSF13 | | -1.07 | | 1.10E-01 |
|  |  | TNFRSF13B | | -1.02 | | 7.51E-01 |
|  |  | TNFRSF11A | | 1.47 | | 3.03E-04 |
|  |  | TNFSF9 | | -1.01 | | 8.60E-01 |
|  |  | TNFSF14 | | -1.05 | | 3.35E-01 |
|  |  | TNFRSF13C | | 1.03 | | 2.31E-01 |
|  |  | TNFRSF21 | | -1.44 | | 3.08E-05 |
|  |  | TNFRSF10C | | -1.07 | | 2.75E-01 |
|  |  | TNFRSF10B | | 3.36 | | 1.44E-07 |
|  |  | TNFRSF10D | | 17.29 | | 9.81E-10 |
|  |  | TNFRSF10A | | -1.23 | | 8.89E-02 |
|  |  | TNFRSF11B | | -10.14 | | 1.82E-09 |
|  |  | TNFSF15 | | -1.31 | | 4.70E-03 |
|  |  | TNFSF8 | | 1.00 | | 9.51E-01 |
|  |  | TNF | | -1.09 | | 3.01E-01 |
|  |  | TNF | | -1.09 | | 3.01E-01 |
|  |  | TNF | | -1.09 | | 3.01E-01 |
| ***TRADD*** | | TRADD | | -1.78 | | 5.38E-06 |
| ***FADD*** | | FADD | | -1.30 | | 2.60E-03 |
| ***Caspase*** | | CASP12 | | -1.05 | | 3.66E-01 |
|  |  | CASP4 | | -1.59 | | 2.51E-04 |
|  |  | CASP5 | | -1.02 | | 5.16E-01 |
|  |  | CASP1 | | -1.05 | | 4.05E-01 |
|  |  | CASP14 | | 1.01 | | 8.35E-01 |
|  |  | CASP10 | | 2.03 | | 1.71E-05 |
|  |  | CASP8 | | -2.05 | | 4.67E-05 |
|  |  | CASP6 | | -1.34 | | 7.05E-04 |
|  |  | CASP3 | | 3.42 | | 4.78E-07 |
|  |  | CASP2 | | 1.11 | | 1.73E-01 |
| ***BID*** | | BID | | -1.54 | | 9.26E-04 |
