# Supplementary material for: MEK5/ERK5 Signaling Suppresses Estrogen Receptor Expression and Promotes Hormone-Independent Tumorigenesis
Source: PLoS One. 2013 Aug 9;8(8):e69291. doi: 10.1371/journal.pone.0069291 (PMC3739787; doi:10.1371/journal.pone.0069291)
Supplement: Table S4 — Altered ER mediated gene expression in MEK5 expressing cells. (DOCX) [file pone.0069291.s008.docx]

**Supplemental Table 4. ER Mediated Gene Expression Changes Associated with MEK5 Overexpression**

| *Gene Symbol* | *Fold Change (Mek5 vs. VEC )* | *p-value* |
| --- | --- | --- |
| MASP1 | -1 | 9.62E-01 |
| PGF | 2.7 | 1.31E-06 |
| KIAA1128 | -1.31 | 1.17E-03 |
| FST | 1.18 | 2.74E-02 |
| ATP1B4 | -1.02 | 7.47E-01 |
| REST | -1.26 | 1.36E-03 |
| SART3 | -1.42 | 2.38E-04 |
| MXI1 | 2.19 | 1.74E-04 |
| PLEC1 | -2.09 | 4.41E-05 |
| PICALM | -1.45 | 5.90E-05 |
| AGPS | 92.07 | 7.07E-10 |
| WWP1 | -2.57 | 7.71E-07 |
| EIF1AX | 1.55 | 9.01E-05 |
| BTBD3 | 1.29 | 3.19E-04 |
| RARA | -2.08 | 1.85E-05 |
| RAB27B | -12.13 | 3.45E-07 |
| MAP2K7 | 1.15 | 1.23E-02 |
| MAP2K5 | 2.56 | 9.54E-06 |
| COL10A1 | -1.07 | 8.55E-02 |
| WNT10A | -1.11 | 8.01E-02 |
| BRAF | 1.71 | 3.32E-05 |
| ROCK2 | -1.25 | 1.80E-04 |
| RCOR2 | 1.26 | 7.06E-03 |
| BARHL1 | -1.05 | 2.99E-01 |
| RCOR1 | -2.75 | 2.63E-06 |
| ZNF503 | 3.37 | 4.96E-06 |
| SAG | -1.09 | 6.64E-02 |
| LETM1 | -1.23 | 8.43E-02 |
| CD37 | -1.06 | 1.48E-01 |
| ADAMTS8 | 1.14 | 1.79E-01 |
| MAPK6 | -3.42 | 3.97E-06 |
| SMPX | 1.1 | 3.90E-01 |
| PMP22 | -2.5 | 1.25E-06 |
| GLG1 | -1.08 | 1.90E-01 |
| ALDOA | -1.24 | 3.65E-03 |
| NFKBIA | -3.54 | 7.88E-08 |
| ARF5 | 1 | 9.35E-01 |
| KCNJ2 | 1.35 | 4.25E-04 |
| ADCYAP1 | 1.01 | 3.84E-01 |
| GAD2 | 1.05 | 2.69E-01 |
| MAML3 | -1.2 | 2.99E-02 |
| DNMT3A | -1.14 | 6.50E-03 |
| ESRRB | 1.15 | 6.89E-02 |
| CUGBP1 | -1.37 | 5.04E-04 |
| EME1 | 1.24 | 7.01E-03 |
| TREX2 | -1.03 | 6.27E-01 |
| ESRRG | 2.92 | 1.82E-04 |
| KIAA0664 | -1.45 | 2.35E-03 |
| NKX6-1 | 1.13 | 1.07E-01 |
| PMFBP1 | 1.35 | 1.26E-03 |
| TRPS1 | -2.65 | 2.49E-06 |
| SCHIP1 | 4.2 | 3.08E-07 |
| KCNJ15 | -1.11 | 1.26E-02 |
| PPARA | 2.93 | 5.24E-05 |
| GFAP | 1.01 | 8.18E-01 |
| JPH2 | -1.06 | 2.84E-01 |
| SLC20A1 | 1.12 | 2.16E-01 |
| FGF9 | 7.09 | 5.64E-07 |
| GREB1 | -24.88 | 3.20E-09 |
| CPEB3 | 1.67 | 1.27E-04 |
| DAXX | 1.28 | 8.65E-04 |
| KCNIP4 | -1.11 | 4.54E-02 |
| CAMKK2 | -1.19 | 9.00E-03 |
| MAP1LC3A | -1.08 | 2.06E-01 |
| SLC25A3 | -1.3 | 1.22E-03 |
| USP12 | 1.03 | 5.03E-01 |
| SLC25A1 | -1.09 | 1.11E-01 |
| NR2F2 | -1.21 | 2.12E-02 |
| NR2F1 | 4.37 | 9.28E-07 |
| RAP2C | -1.19 | 1.12E-03 |
| RASIP1 | -1.04 | 6.38E-01 |
| MYADM | -2.75 | 2.71E-06 |
| CHRDL1 | 1.07 | 1.89E-01 |
| CKM | -1.06 | 1.13E-01 |
| SEMA4G | 1.48 | 4.30E-03 |
| DACT2 | -1.06 | 3.70E-01 |
| SLC26A9 | -1.05 | 4.00E-01 |
| WNT9B | -1.11 | 2.01E-01 |
| GNAS | -1.5 | 2.42E-05 |
| STEAP2 | 9.76 | 1.83E-06 |
| DOLPP1 | 1.05 | 6.64E-02 |
| CLOCK | -1.48 | 3.30E-03 |
| KCNH5 | -1.02 | 6.82E-01 |
| SCML4 | 1.05 | 3.76E-01 |
| COX7A2L | -1.72 | 4.77E-05 |
| CIC | -1.17 | 1.32E-01 |
| ZFP36L1 | -3.71 | 1.69E-07 |
| RB1CC1 | 1.66 | 6.21E-04 |
| SLC4A7 | -2.98 | 5.70E-08 |
| BCL6 | -1.59 | 8.48E-04 |
| PRKAA2 | 7.71 | 2.30E-07 |
| TLX3 | 1.44 | 1.34E-03 |
| RASA4 | 1.29 | 1.00E-02 |
| WNT8A | 1.07 | 1.79E-01 |
| SPATA7 | -2.07 | 1.31E-03 |
| WNT8B | -1.01 | 7.00E-01 |
| TBX6 | -1.16 | 7.08E-02 |
| NLK | 1.05 | 8.38E-02 |
| NLGN2 | 1.01 | 9.37E-01 |
| ELAVL4 | 1.02 | 7.01E-01 |
| MID1 | 17.27 | 7.79E-08 |
| DKK2 | 1.09 | 1.79E-01 |
| DUSP4 | -5.56 | 2.49E-07 |
| TUBA8 | 1.2 | 4.37E-03 |
| DUSP3 | -1.07 | 1.58E-03 |
| MEOX2 | 1.01 | 8.40E-01 |
| CDH16 | -1.07 | 3.55E-01 |
| SLC16A6 | -8.92 | 1.61E-06 |
| MRPL27 | -1.07 | 2.25E-01 |
| GFPT2 | 2.69 | 1.58E-05 |
| ATP5A1 | 1.37 | 1.42E-04 |
| ATP6V0A4 | -1.98 | 1.01E-03 |
| PDGFB | -1.63 | 1.11E-04 |
| F13A1 | -1.05 | 9.52E-02 |
| ANKRD2 | -1.08 | 2.34E-01 |
| UBQLN1 | -1.02 | 4.64E-01 |
| FLJ10404 | -1.02 | 2.39E-01 |
| ZIC2 | 2.6 | 1.34E-05 |
| IFRG15 | -1.2 | 7.79E-02 |
| ATOH1 | -1.03 | 7.78E-01 |
| WNT4 | -1.09 | 3.27E-02 |
| MLL5 | -1.62 | 3.15E-06 |
| WNT3 | 1.14 | 2.07E-02 |
| PGLYRP2 | -1.14 | 1.58E-01 |
| LOX | 10.09 | 8.60E-08 |
| CHAT | -1.1 | 2.45E-01 |
| SATB1 | 27.81 | 2.48E-09 |
| SMCR8 | -1.39 | 6.44E-04 |
| NUDT4 | -1.36 | 5.32E-02 |
| SOCS2 | 1.41 | 4.09E-06 |
| YTHDF3 | -1.08 | 6.60E-02 |
| COX6C | -2.49 | 2.15E-05 |
| ISCU | -1.26 | 1.32E-02 |
| RASGRF1 | -1.11 | 6.81E-02 |
| FLJ41603 | -1.06 | 3.79E-01 |
| TFAP2C | -13.5 | 1.69E-09 |
| WNT5A | 5.96 | 1.86E-07 |
| SLC38A2 | -1.31 | 7.08E-04 |
| EEF1B2 | 1.1 | 3.18E-01 |
| FBXO40 | -1.02 | 6.34E-01 |
| BMPR2 | -1.37 | 1.04E-03 |
| OAZ2 | 1.31 | 7.72E-04 |
| OAZ3 | -1.56 | 7.24E-04 |
| FLJ40125 | 1.22 | 8.46E-02 |
| TTBK2 | 1.87 | 4.52E-06 |
| HOXA5 | 9.76 | 4.28E-08 |
| ITGB8 | -1.12 | 1.94E-01 |
| OVOL1 | -5.8 | 2.12E-06 |
| TEF | -1.1 | 1.59E-01 |
| NDRG2 | 1.08 | 7.01E-02 |
| HCN4 | 1.01 | 1.92E-01 |
| LRFN4 | -1.7 | 8.76E-05 |
| LPL | 1.92 | 8.65E-05 |
| GABARAPL2 | 1.27 | 1.59E-02 |
| B4GALT3 | 1.15 | 2.27E-02 |
| EEF1A2 | -3.14 | 4.13E-07 |
| TOMM40 | -1.51 | 2.32E-06 |
| AK2 | 1.46 | 1.97E-06 |
| GCM1 | -1.17 | 2.17E-02 |
| PHF1 | -1.44 | 5.12E-05 |
| SLC7A3 | 2.57 | 1.40E-06 |
| CPNE1 | -1.21 | 6.71E-03 |
| DNAJB4 | 3.03 | 6.70E-08 |
| ABL1 | 1.21 | 6.47E-02 |
| CDC14A | -1.35 | 2.96E-03 |
| MRVI1 | 1.03 | 1.19E-01 |
| VGF | -1.54 | 1.92E-03 |
| GPC4 | 4.65 | 2.85E-06 |
| BZW2 | -1.29 | 1.51E-02 |
| SPRY2 | 4.62 | 1.29E-05 |
| RANBP9 | 1.32 | 8.92E-05 |
| SOX15 | -1.04 | 6.63E-01 |
| LOXL4 | 1.37 | 4.28E-03 |
| LOXL3 | -1.51 | 2.30E-03 |
| NDUFS1 | -1.09 | 8.10E-02 |
| PIGW | 1.25 | 4.46E-02 |
| GALNTL2 | -1.04 | 2.17E-01 |
| LDB2 | -1.32 | 1.21E-03 |
| ATP6V1A | -1.05 | 1.78E-01 |
| COMMD3 | -1.25 | 4.60E-02 |
| ERN1 | -1.36 | 1.06E-03 |
| CA4 | -1.32 | 1.89E-03 |
| JMJD1C | -1.15 | 2.57E-04 |
| AOC2 | -1.29 | 1.30E-02 |
| LCP1 | -7.64 | 9.94E-08 |
| ROM1 | -1.28 | 1.62E-02 |
| FKBP4 | -1.67 | 2.57E-06 |
| TCF7L2 | 1.43 | 1.31E-04 |
| GPR3 | -1.06 | 3.98E-01 |
| TPM3 | -1.5 | 1.36E-05 |
| UBE2R2 | -1.18 | 4.21E-02 |
| PRDM13 | 1.1 | 9.49E-02 |
| PSMF1 | -1.46 | 4.51E-04 |
| SLC35B1 | -1.39 | 4.57E-03 |
| NPAS2 | -1.09 | 1.02E-01 |
| DCX | -1.02 | 7.50E-01 |
| CHD6 | -2.35 | 5.16E-07 |
| CNNM2 | 1.2 | 2.91E-02 |
| B3GALT2 | -1.17 | 1.14E-01 |
| PLAC1 | 2.16 | 2.78E-04 |
| MAP1A | 1.18 | 1.20E-01 |
| SLC10A2 | -1.05 | 2.72E-01 |
| XPNPEP1 | 2.15 | 3.39E-07 |
| INVS | 1.03 | 5.83E-01 |
| KCNN2 | -1.04 | 3.64E-01 |
| VPS24 | -1.56 | 2.31E-04 |
| SP6 | -1.37 | 1.37E-04 |
| SPTAN1 | -1.08 | 3.95E-01 |
